# Molecular Characterization of Four Alkaline Chitinases from Three Chitinolytic Bacteria Isolated from a Mudflat

**DOI:** 10.3390/ijms222312822

**Published:** 2021-11-26

**Authors:** Sung Kyum Kim, Jong Eun Park, Jong Min Oh, Hoon Kim

**Affiliations:** 1 Department of Agricultural Chemistry, Sunchon National University, Suncheon 57922, Korea; kimsk0851@scnu.ac.kr; 2 Department of Pharmacy, and Research Institute of Life Pharmaceutical Sciences, Sunchon National University, Suncheon 57922, Korea; 1200113@s.scnu.ac.kr (J.E.P.); ddazzo005@naver.com (J.M.O.)

**Keywords:** *Aeromonas* sp., *Chitinibacter* sp., gene cluster, alkaline chitinases, endochitinases, exochitinase, chitooligosaccharides

## Abstract

Four chitinases were cloned and characterized from three strains isolated from a mudflat: *Aeromonas* sp. SK10, *Aeromonas* sp. SK15, and *Chitinibacter* sp. SK16. In SK10, three genes, Chi18A, Pro2K, and Chi19B, were found as a cluster. Chi18A and Chi19B were chitinases, and Pro2K was a metalloprotease. With combinatorial amplification of the genes and analysis of the hydrolysis patterns of substrates, Chi18A and Chi19B were found to be an endochitinase and exochitinase, respectively. Chi18A and Chi19B belonged to the glycosyl hydrolase family 18 (GH18) and GH19, with 869 and 659 amino acids, respectively. Chi18C from SK15 belonged to GH18 with 864 amino acids, and Chi18D from SK16 belonged to GH18 with 664 amino acids. These four chitinases had signal peptides and high molecular masses with one or two chitin-binding domains and, interestingly, preferred alkaline conditions. In the activity staining, their sizes were determined to be 96, 74, 95, and 73 kDa, respectively, corresponding to their expected sizes. Purified Chi18C and Chi18D after pET expression produced *N*,*N*′-diacetylchitobiose as the main product in hydrolyzing chitooligosaccharides and colloidal chitin. These results suggest that Chi18A, Chi18C, and Chi18D are endochitinases, that Chi19B is an exochitinase, and that these chitinases can be effectively used for hydrolyzing natural chitinous sources.

## 1. Introduction

Chitin—an insoluble homopolymer of β-(1,4)-linked *N*-acetylglucosamine—is the second most abundant biopolymer in nature [1] and is also a major component of the exoskeleton of arthropods and mold cell walls. Chitin-degrading enzymes are classified into two categories: endochitinases and exochitinases. Endochitinases (EC 3.2.1.14) are enzymes that randomly cleave the internal β-(1,4)-linkages of *N*-acetylglucosamine in chitin and chitodextrins, while exochitinases, β-N-acetylhexosaminidases (or β-N-acetylglucosaminidases) (EC 3.2.1.52) are enzymes that hydrolyze non-reducing terminal N-acetyl-D-hexosamine residues in *N*-acetyl-β-D-hexosaminides (usually glucose or galactose) [2]. Traditionally, two groups of exochitinases, such as chitobiosidases (EC 3.2.1.29) and β-N-acetylglucosaminidases (EC 3.2.1.30), have been incorporated into the category β-N-acetylhexosaminidases (EC 3.2.1.52).

Chitinases belong to the glycosyl hydrolase (GH) families 18, 19, and 20 [3]. GH18 and GH19 are mainly endochitinases, and GH20 includes *N*-acetyl-β-hexosaminidase. GH18 chitinases have been found in various organisms from bacteria to humans, including plants, molds, and vertebrates, while GH19 chitinases are found mainly in plants and are not common in bacteria, having recently been found in only a few strains, such as *Chitinophaga* sp. [4], *Pseudoalteromonas rubra*, [5], and *Streptomyces alfalfa* [6].

Chitinases can have various applications, for instance, in waste management and as biocontrol agents, as well as in medicine (e.g., antifungal agents) and in food industries [7]. Bacterial chitinases have been isolated from various strains, including metagenomes [8,9,10], especially from marine sources, and have been used in different biotechnological fields [2,11]. Recently, chitinase genes were isolated and cloned from several marine bacteria, such as *Paenibacillus barengoltzii* [12], *Photobacterium galatheae* S2753, *Pseudoalteromonas piscicida* S2040 and S2724 [13], *Streptomyces thermodiastaticus* [14], *Streptomyces albolongus* [15], *Vibrio* sp. [16], *Myxococcus fulvus* [17], and *P. rubra* [5]. Mudflats are habitats supporting large numbers of highly diverse life forms, including chitin-degrading microorganisms. Three novel chitin-degrading microorganisms, SK10, SK15, and SK16, were isolated from a mudflat in Suncheon Bay, South Korea, of which SK16 was identified and named *Chitinibacter suncheonensis* sp. nov. [18]. In this study, SK10 and SK15 were identified as *Aeromonas* species. No information has been available about chitinase cloning from *Chitinibacter* sp., except for a chitinase from *Chitinibacter tainanensis* [19], and only a few chitinases from *Aeromonas* sp. have been reported, such as *Aeromonas caviae* [20] and *A. caviae* CB101 [21,22].

Furthermore, it has been reported that bacterial chitinases are optimally active in acidic conditions; alkalophilic chitinases are very rare [6,23]. In this study, four alkalophilic chitinase genes (*chi*18A, *chi*19B, *chi*18C, and *chi*18D) were cloned from the three isolates; three GH18 and one GH19 family chitinases were identified, and the encoded chitinases were characterized. Among them, two endochitinase genes (*chi*18C and *chi*18D) were cloned into a pET expression vector and purified using an affinity column, and their enzymatic properties were characterized.

## 2. Results

### 2.1. Isolation of Chitinolytic Bacteria

**Figure 1:**
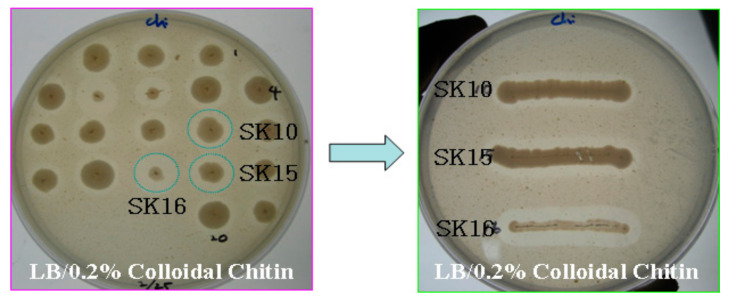
Growth and chitinolytic activities of the chitin-degrading microorganisms isolated from mudflats on LB containing 0.2% colloidal chitin (LBCC) agar plates. Each strain was streaked on LBCC agar plates and incubated at 30 °C for three days.

**Figure 2:**
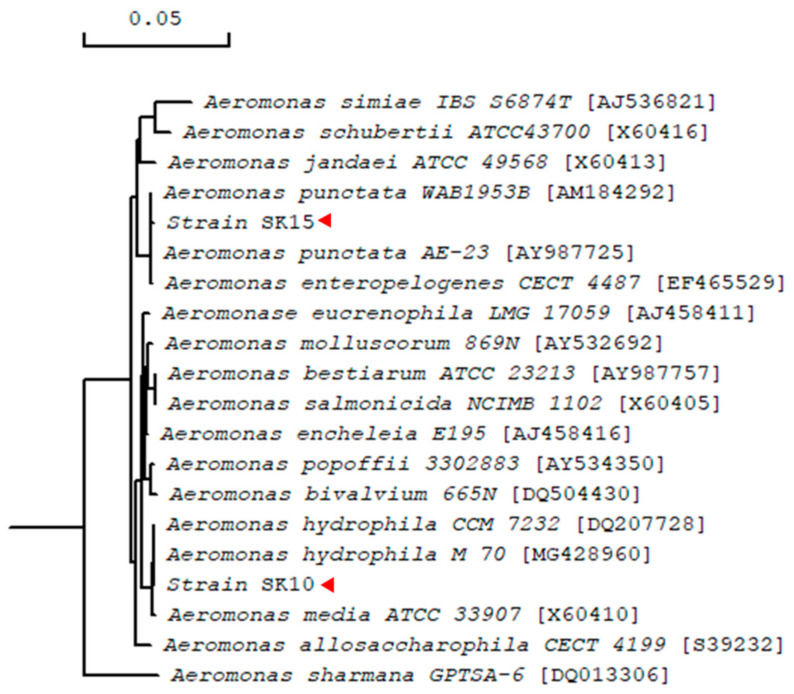
Phylogenetic tree of *Aeromonas* isolates SK10 and SK15 based on their 16S rRNA sequences. The phylogenetic tree was constructed using observed divergence method with bootstrap trials of 1000 in DNA/MAN.

From the enriched mudflat sample, 20 colonies were primarily selected on LBCC agar plates. Based on the color and shape of the colony and the size of the clear zone, six colonies were further selected. After measuring the extracellular chitinase activity of the six colonies, three colonies were selected for further experiments: SK10, SK15, and SK16 (Figure 1). Chitinase activities of the smallest colonies with the largest zone were low in culture broth assays. The 16S rRNA sequence of the isolate SK10 showed 100% similarity with *Aeromonas hydrophila* (GenBank accession number MG428960), and that of SK15 showed 99.9% similarity with *Aeromonas punctata* (AM184292) in the NCBI server. That of SK16 showed 98.2% similarity with *C. tainanensis* (AY264287) and represented a novel species of the genus *Chitinibacter*, i.e., *C. suncheonensis* sp. nov. [18]. The isolates SK10 and SK15 were named *Aeromonas* sp. SK10 and *Aeromonas* sp. SK15, respectively, and their phylogenetic tree is shown in Figure 2.

### 2.2. Growth and Chitinase Production from the Isolates SK10, SK15, and SK16

When the isolates were cultured with shaking in LB, SK10 and SK15 grew faster than SK16, and all three isolates reached a plateau for growth after 12 h (Figure S1). When the isolates were cultured with media, such as LB, LBCC, and 0.5 × LB/colloidal chitin, chitinase activities were maximal at 27 or 30 h of culture in an LBCC medium (Figure S1).

### 2.3. Growth and Chitinase Production of the Isolates SK10, SK15, and SK16

**Figure 3:**
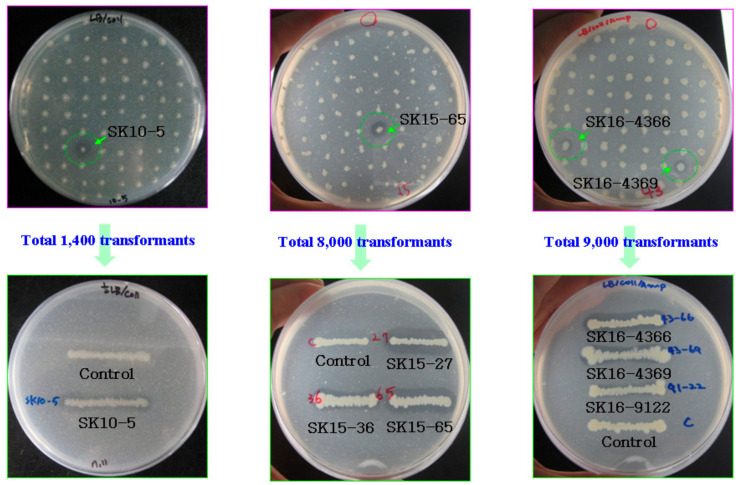
Screening of chitinase-positive clones using LBCC/ampicillin plates. The clones were streaked on LBCC/ampicillin plates and incubated for three days at 30 °C. The positive clones were streaked on the same plates and compared with a control, which was transformed with a pUC19 vector.

Using the method described in the materials and methods, ~1400, 8000, and 9000 transformants were constructed from SK10, SK15, and SK16 genomes. From the libraries, one active clone (SK10-5) from SK10, three active clones (SK15-27, SK15-36, and SK15-65), and three active clones (SK16-4366, SK16-4369, and SK16-9122) were obtained (Figure 3). The recombinant plasmids of the clones had insert DNA fragments of >3.0 kb in length estimated by restriction enzyme digestion.

#### 2.3.1. Cloning of Chitinases from SK10

**Figure 4:**
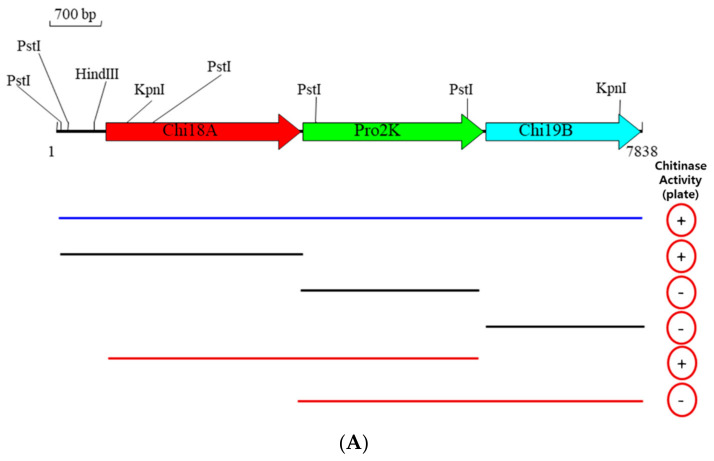

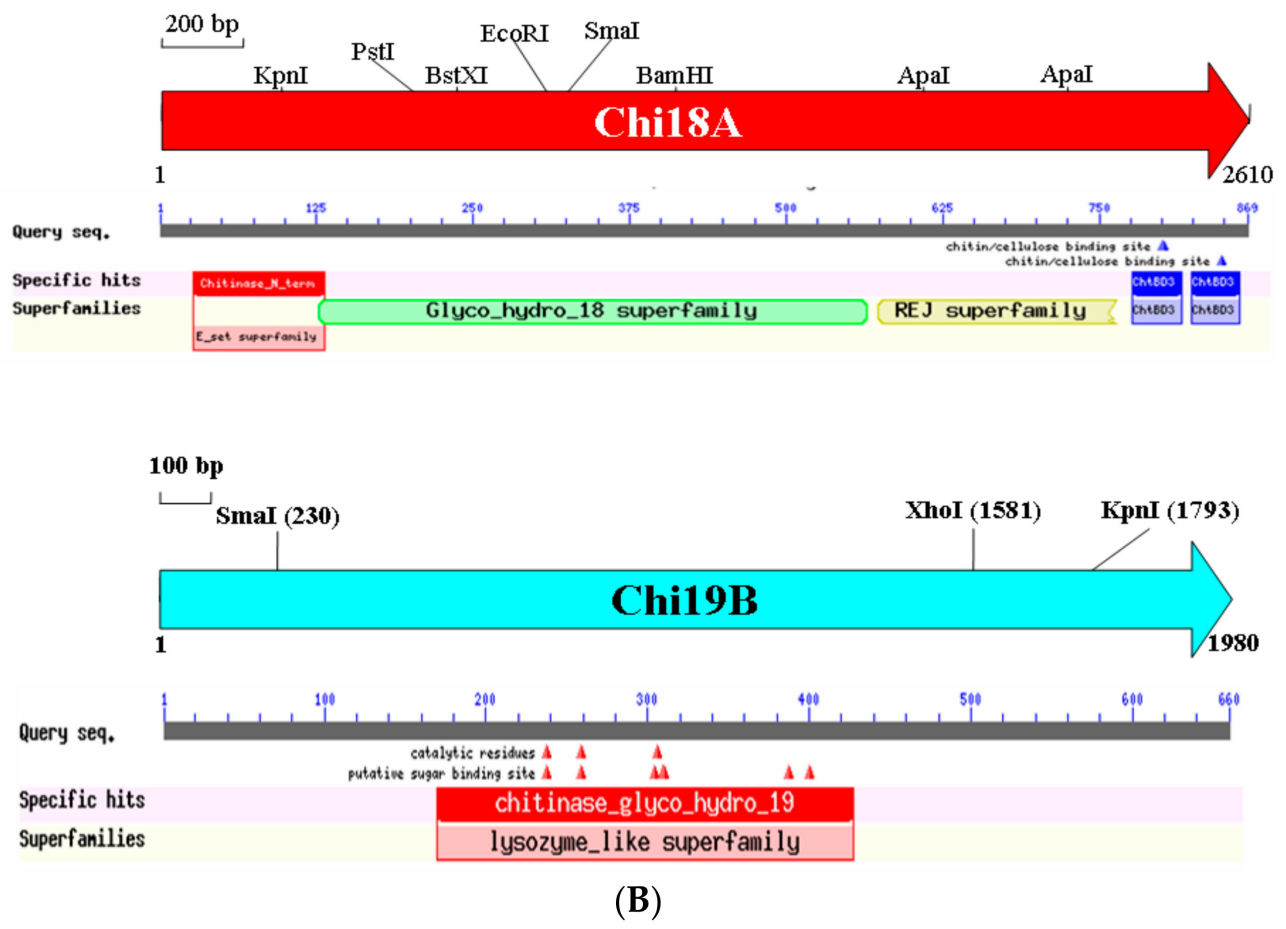
Physical maps of the insert DNA of SK10-5 (**A**), and Chi18A and Chi19B (**B**).

The insert DNA of SK10-5 was 7838 bp in length determined by nucleotide sequencing and comprised three ORFs: ORF1 (2610 bp); ORF2 (2427 bp); and ORF3 (1980 bp), encoding proteins of 869, 808, and 659 amino acid residues in length, respectively (Figure 4A). The three encoded proteins were matched to chitinases belonging to the glycosyl hydrolase family 18 (GH18), metalloproteases, and chitinases belonging to GH19, respectively, and named Chi18A, Pro2K, and Chi19B. Chi18A contained a chitin-binding domain (ChBD) at the C-terminus, and Chi19B also belonged to a lysozyme-like superfamily (Figure 4B).

Chi18A had a putative signal peptide comprising 23 amino acid residues and a conserved motif of 307FDGVDIDWE315, which was highly conserved in the GH18 chitinases (Figure S2). Chi19B had a signal peptide comprising 42 amino acid residues and conserved motifs that were highly maintained in the GH19 chitinases (Figure S3).

**Figure 5:**
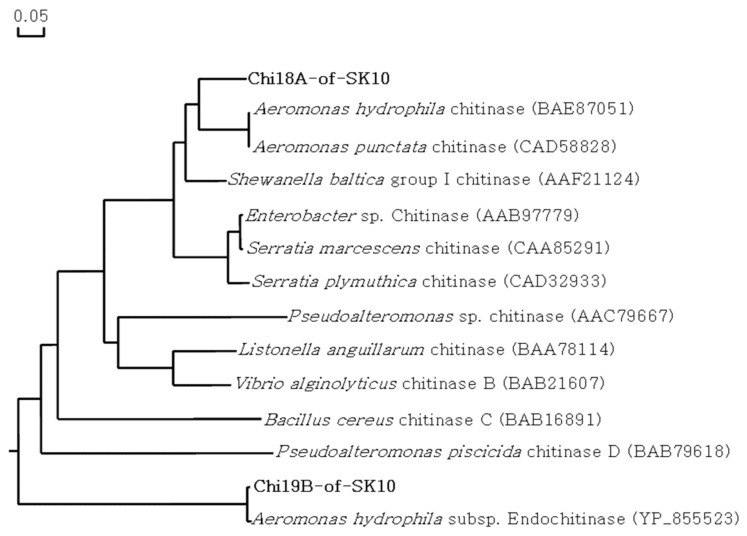
Phylogenetic tree of the Chi18A and Chi19B proteins. The phylogenetic tree shows the evolutionary relatedness and levels of homology between the chitinolytic enzymes. The phylogenetic tree was constructed using the maximum likelihood method with bootstrap trials of 1000 in DNA/MAN.

Chi18A, Chil19B, and Pro2K showed 81.3%, 98.6%, and 98.5% similarities with chitinase from *A. punctata* (BAE87051), endochitinase from *A. hydrophila* (YP_855523), and metalloprotease from *A. hydrophila* (WP_044799315), respectively. In the phylogenetic tree, Chi18A and Chi19B were located at different branches (Figure 5).

#### 2.3.2. Subcloning of Chi18A, Chi19B, and Pro2K from SK10-5

**Figure 6:**
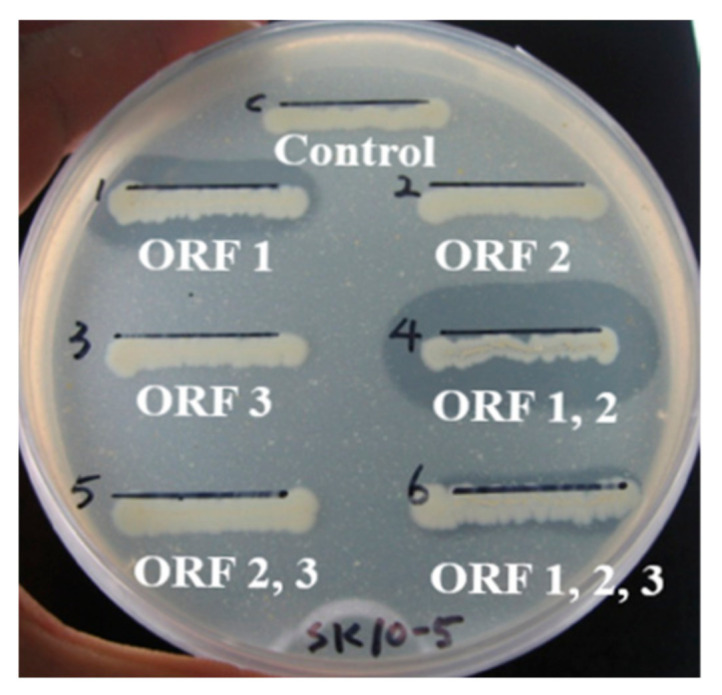
Chitinase activities of subclones of three ORFs in clone SK10-5 on an LBCC plate. ORF1, ORF2, and ORF3 encoded Chi18A, Pro2K, and Chi19B, respectively.

Three ORFs of the clone SK10-5 were subcloned by amplification of the genes individually or combinatorially using the primer sets in Table 1, resulting in six subclones: ORF1, ORF2, ORF3, ORF(1+2), ORF(2+3), and ORF(1+2+3). Among them, ORF1, ORF(1+2), and ORF(1+2+3) showed clear zones on an LBCC agar plate, whereas no clear zones were observed in ORF2, ORF(2+3), and ORF3 (Figure 6). The results are shown in Figure 4A. The size of a clear zone of ORF(1+2) was ~2 times larger than one of ORF1, and a clear zone of ORF(1+2+3) was the smallest among them, based on the distance from the center of each streak. In the liquid assay, ORF(1+2) showed 6.2 times higher chitinase activity than ORF1. These results suggest that Chi18A produced from ORF1 was an endochitinase, and Chi19B produced from ORF3 was an exochitinase. Interestingly, Pro2K, located between Chi18A and Chi19B, was part of an operon. Furthermore, it is likely that Pro2K increased chitinase activity, based on the results of comparing ORF1 (i.e., Chi18A) to ORF(1+2) (i.e., Chi18A/Pro2K). However, adding ORF1 and ORF2 clones separately showed chitinase activity like ORF1 (data not shown). The study about Pro2K’s role will be needed in the future during transcription and translation of Chi18A.

#### 2.3.3. Cloning of Chitinase from SK15

**Figure 7:**
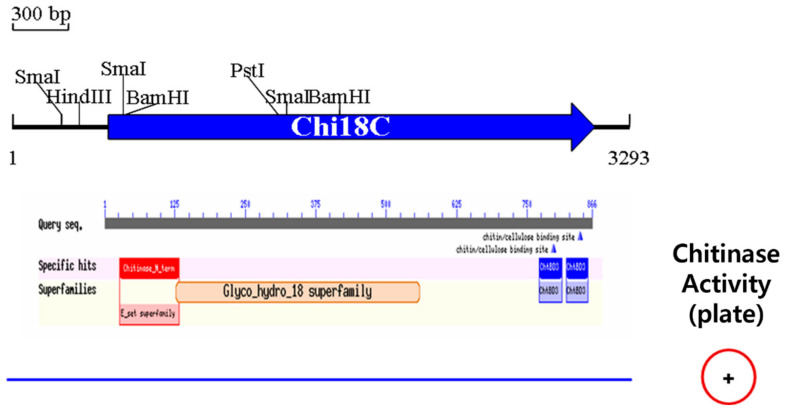
Physical map of the insert DNA of SK15-36. The figure below of the physical map shows the result of BLASTp for Chi18C. The blue line and + symbol at the bottom show DNA of the clone and chitinase activity on the LBCC agar plates, respectively.

**Figure 8:**
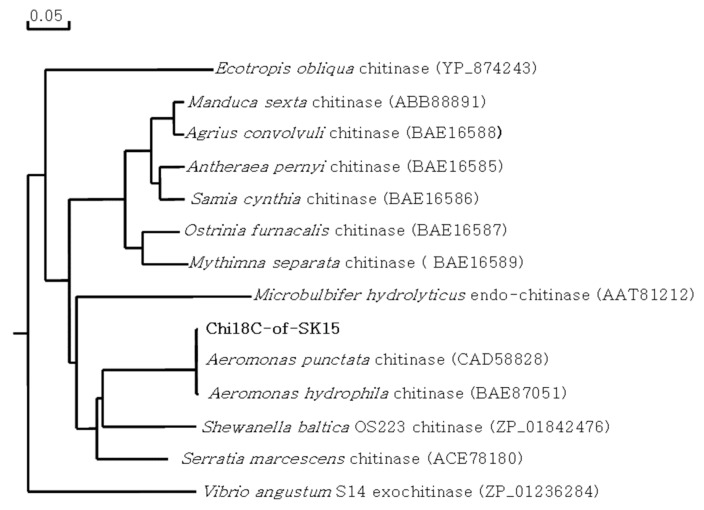
Phylogenetic tree of Chi18C. The phylogenetic tree was constructed using the maximum likelihood method with bootstrap trials of 1000 in DNA/MAN.

In the library, three active clones, SK15-27, SK15-36, and SK15-65, were screened on the plate. Insert DNA of SK15-36 was 3293 bp in length and contained an ORF, matching a chitinase belonging to GH18, named Chi18C (Figure 7). Chi18C comprised 864 amino acid residues with a signal peptide of 23 amino acid residues (Figure S4) and two chitin-binding sites at the C-terminus (Figure 7). Chi18C showed 99.8% similarity with a chitinase from *A. punctata* and was located with other chitinases from *Aeromonas* sp. in the phylogenetic tree (Figure 8). Using a primer set amplifying the conserved motif, it was observed that the same size of DNA fragment appeared in the other two active clones, suggesting that the three clones produced the same kind of chitinase.

#### 2.3.4. Cloning of Chitinase from SK16

**Figure 9:**
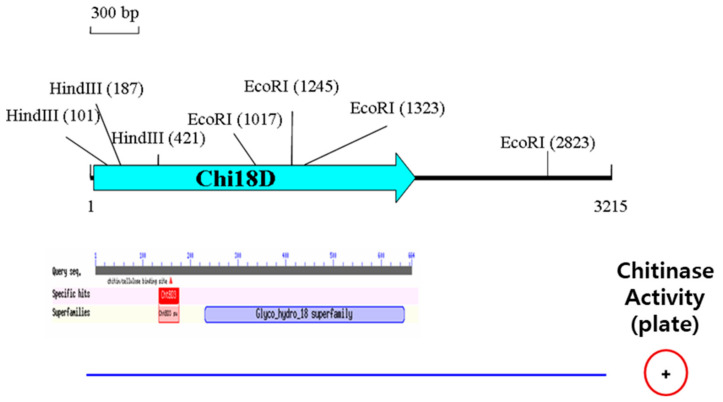
Physical map of the insert DNA of SK16-4366. The result of BLASTp for Chi18D is shown below the physical map. The blue line and + symbol at the bottom represent the DNA of the clone and chitinase activity on the LBCC agar plates, respectively.

**Figure 10:**
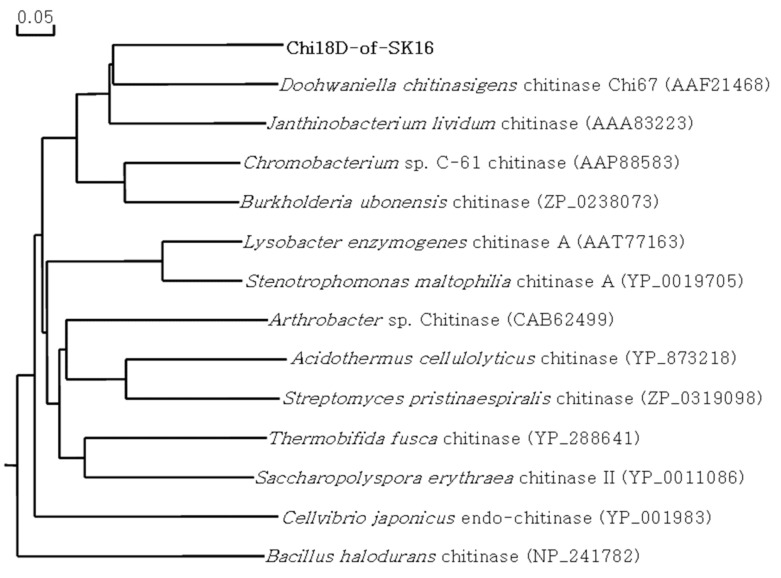
Phylogenetic tree of the Chi18D protein. The phylogenetic tree was constructed using the maximum likelihood method with bootstrap trials of 1000 in DNA/MAN.

In the library, three active clones, SK16-4366, SK16-4369, and SK16-9122, were screened on the plate. The insert DNA of SK16-4366 was 3215 bp in length and contained an ORF, matching a chitinase belonging to GH18, named Chi18D (Figure 9). Chi18D comprised 664 amino acid residues with a signal peptide of 31 amino acid residues and the conserved GH18 motif (Figure S5). Chi18D showed 54.6% similarity (the highest level of similarity) with a chitinase from *Doohwaniella chitinasigens* and was located close to chitinase in the phylogenetic tree (Figure 10). Using a primer set amplifying the conserved motif, it was observed that the same size of DNA fragment appeared in the other two active clones, suggesting that the three clones produced the same kind of chitinase.

### 2.4. Biochemical Properties of Chitinases in Crude Extracts

#### 2.4.1. Chi18A

**Figure 11:**
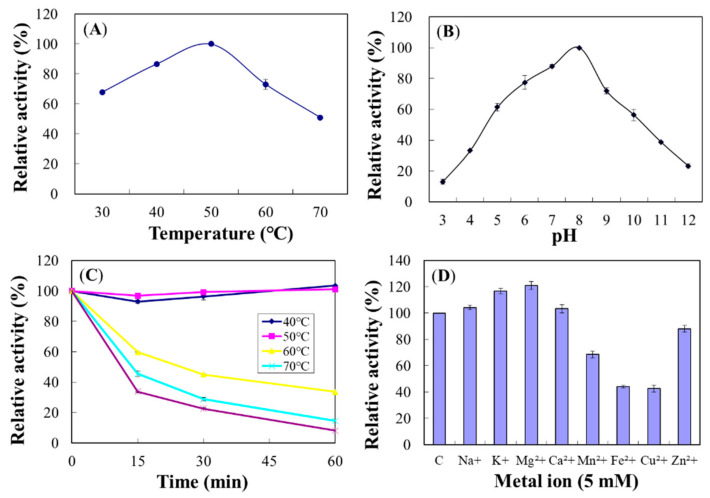
Effects of temperature (**A**), pH (**B**), thermostability (**C**), and metal ions (**D**) on the enzyme activity of Chi18A. The properties were characterized using the DNS method with colloidal chitin as a substrate.

The optimum temperature for Chi18A was 50 °C (Figure 11A), and activity was decreased at 70 °C to a half of the maximum. The optimum pH for Chi18A was 8.0 (Figure 11B). Regarding heat stabilities, Chi18A was stable at 50 °C for up to 1 h preincubation but lost >60% of its original activity after 15 min preincubation at 60 °C (Figure 11C). The half-lives of Chi18A at 60 and 70 °C were 25.0 and 13.8 min, respectively.

Cations Mg^2+^ and K^+^ increased Chi18A activity to 120% and 117%, respectively, and Cu^2+^, Fe^2+^, Mn^2+^, and Zn^2+^ decreased its activity to 42.6%, 44.1%, 68.5%, and 88.1%, respectively (Figure 11D). In the experiments on concentration dependency, Mg^2+^ and K^+^ showed maximal activity at 5.0 and 7.5 mM, respectively (Figure S6).

#### 2.4.2. Chi19B

**Figure 12:**
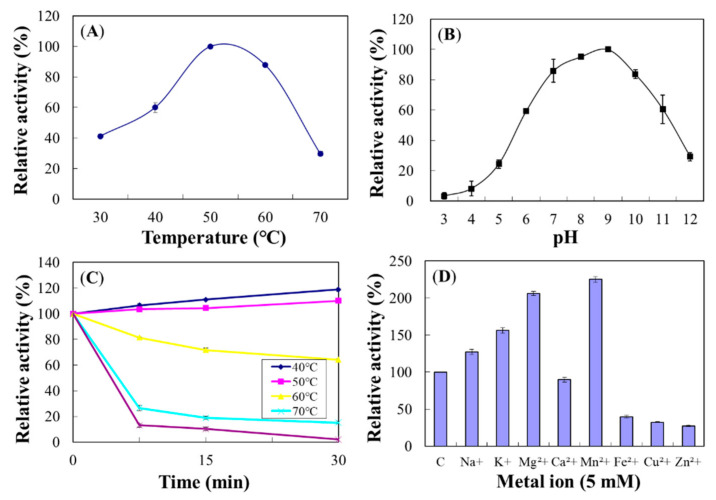
Effects of temperature (**A**), pH (**B**), thermostability (**C**), and metal ions (**D**) on the enzyme activity of Chi19B. The properties were characterized using the DNS method with colloidal chitin as a substrate.

The optimum temperature for Chi19B was 50 °C (Figure 12A), and activity was decreased at 70 °C to 30% of the maximum. The optimum pH for Chi19B was 9.0 (Figure 12B). Regarding heat stabilities, Chi19B was stable at 50 °C for up to 1 h preincubation and lost 35.8% of its original activity after 1 h preincubation at 60 °C, but lost >70% activity after 15 min preincubation at 70–80 °C (Figure 12C). The half-life of Chi19B at 70 °C was 10.2 min.

Cations Mn^2+^, Mg^2+^, K^+^, and Na^+^ increased Chi19B activity to 225.3%, 206.2%, 156.2%, and 127.4%, respectively, and Zn^2+^, Cu^2+^, and Fe^2+^ decreased its activity to 27.4%, 32.5%, and 39.9%, respectively (Figure 12D). In the experiments on concentration dependency, Mn^2+^ and Mg^2+^ showed maximal activity at 2.5 mM, and K^+^ and Na^+^ showed maximal activity at 7.5 mM (Figure S7).

#### 2.4.3. Chi18C

**Figure 13:**
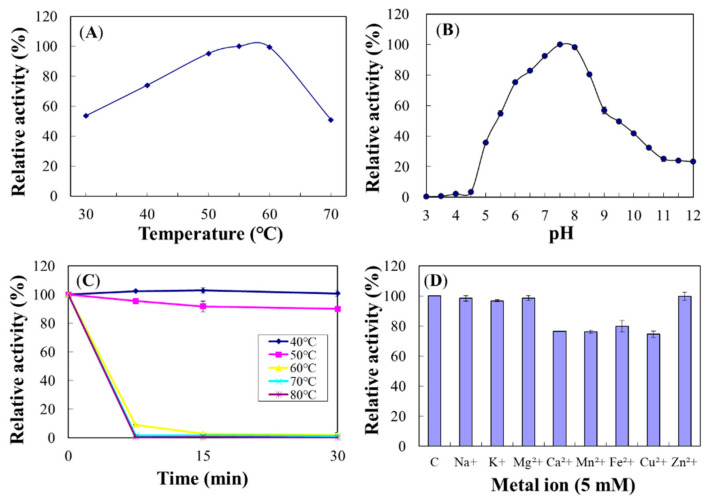
Effects of temperature (**A**), pH (**B**), thermostability (**C**), and metal ions (**D**) on the enzyme activity of Chi18C. The properties were characterized using the DNS method with colloidal chitin as a substrate.

The optimum temperature for Chi18C was 55–60 °C (Figure 13A), and activity was decreased at 70 °C to 50% of the maximum. The optimum pH for Chi18C was 7.0–8.0 (Figure 13B). Regarding heat stabilities, Chi18C was stable at 50 °C for up to 1 h preincubation; however, its activity was drastically lowered, almost to zero, after 15 min preincubation at 60–80 °C (Figure 13C). The half-life of Chi18C at 70 °C was 7.6 min.

No cations tested increased Chi18C activity; however, Cu^2+^, Mn^2+^, Ca^2+^, and Fe^2+^ moderately decreased its activity to 74.5%, 76.1%, 76.5%, and 82.0%, respectively (Figure 13D).

#### 2.4.4. Chi18D

**Figure 14:**
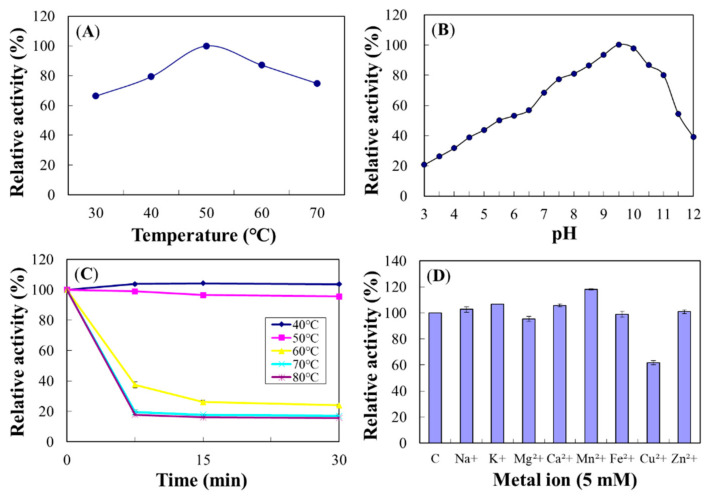
Effects of temperature (**A**), pH (**B**), thermostability (**C**), and metal ions (**D**) on the enzyme activity of Chi18D. The properties were characterized using the DNS method with colloidal chitin as a substrate.

The optimum temperature for Chi18D was 50 °C (Figure 14A), and its activity was decreased at 70 °C to 80% of the maximum. The optimum pH for Chi18D was 9.5 (Figure 14B). Regarding heat stabilities, Chi18D was stable at 50 °C for up to 1 h preincubation; however, it rapidly lost >60% of its activity after 15 min preincubation at 60–80 °C (Figure 14C). The half-life of Chi18D at 70 °C was 9.3 min.

Cation Mn^2+^ increased Chi18D activity to 120%; however, Cu^2+^ decreased its activity to 61.7% (Figure 14D). In the experiments on concentration dependency, Mn^2+^ showed maximal activity at 5.0 mM (Figure S8).

### 2.5. Site-Directed Mutagenesis of Chitinases

**Figure 15:**
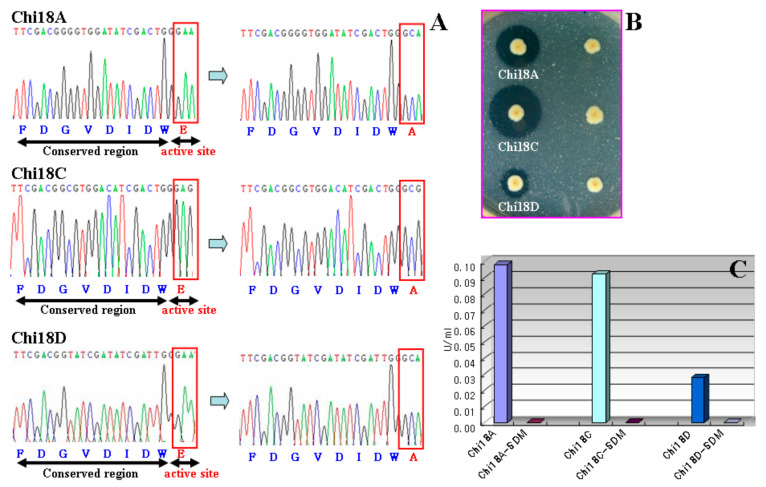
Confirmation of active sites of Chi18A, Chi18C, and Chi18D by site-directed mutagenesis. (**A**) Change of active site by site-directed mutagenesis from GAA and GAG (Glu) to GCA and GCG (Ala). (**B**) Chitinase activities of the mutants on an LBCC plate. (**C**) Chitinase activities of the crude extracts of the mutants.

When Glu315, Glu315, and Glu408 of Chi18A, Chi18C, and Chi18D, respectively, were mutated to Ala, the activities of the mutants were found to be lost completely on an LBCC plate and assay of the crude extract (Figure 15), verifying that those residues were catalytic sites of the enzymes.

### 2.6. Analysis of the Molecular Masses of Chitinases Using Activity Staining

**Figure 16:**
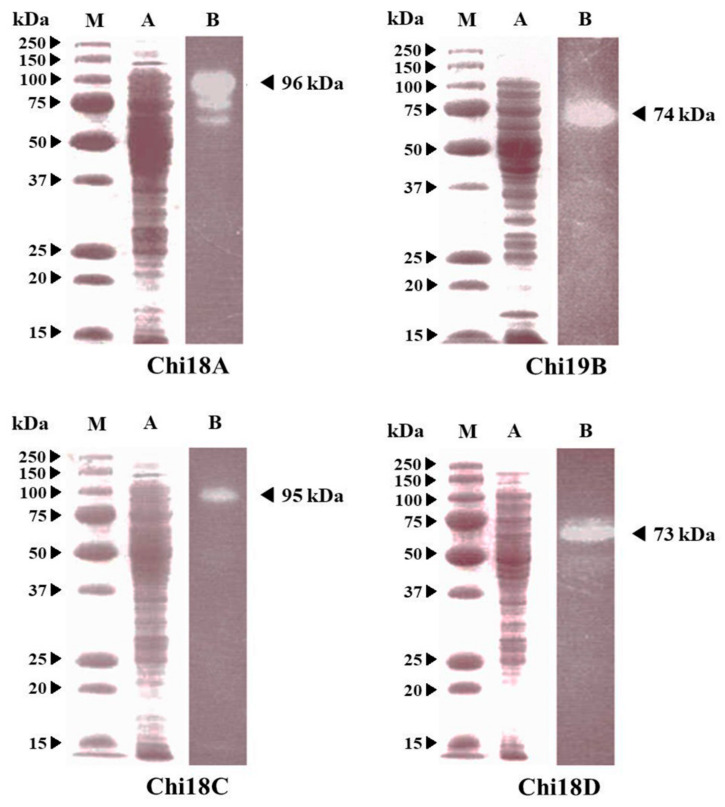
Molecular masses of the chitinases produced by Chi18A, Chi19B, Chi18C, and Chi18D-containing clones. Lanes M, molecular weight standards; A, stained with Coomassie blue after SDS-PAGE; Lanes B, activity stained with MUCh_2_.

By activity staining with MUCh_2_ after SDS-PAGE and the renaturation method, the molecular masses of Chi18A, Chi19B, Chi18C, and Chi18D were estimated to be 96, 74, 95, and 73 kDa, respectively (Figure 16). The results closely corresponded to the expected values. For Chi18A, two active bands, corresponding to 75 and 65 kDa, were detected due to the internal cleavage of the enzyme.

### 2.7. Purification of Chi18C and Chi18D

**Figure 17:**
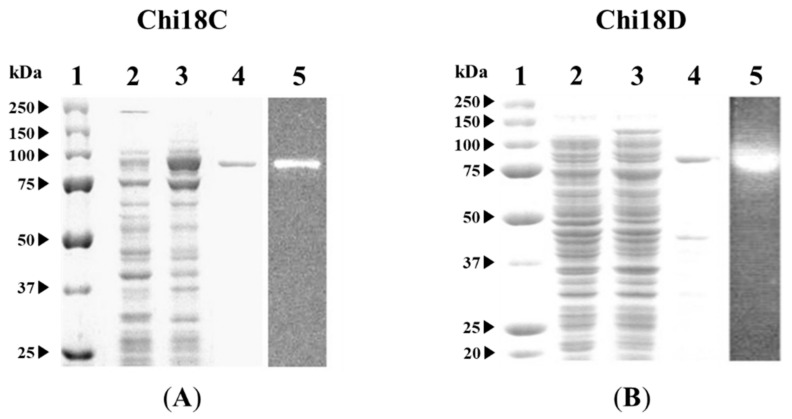
SDS-PAGE (Lanes 1–4) and activity staining after renaturation treatment of chitinase (Lane 5) expressed in *E. coli* BL21 (DE3) harboring pET-28a(+)/*chi*18C (**A**) or/*chi*18D (**B**). Each panel represents: Lane 1, standard marker; Lane 2, the crude extract from each clone; Lane 3, the crude extract from each clone induced by IPTG; Lane 4, the purified chitinase using an Ni-NTA column from each clone; Lane 5, activity staining of the purified chitinase Chi18C or Chi18D from each clone.

Each enzyme was purified using the Ni-NTA method after Chi18C and Chi18D were separately expressed with a pET28a(+) vector. Chi18C was greatly induced by IPTG, purified to homogeneity, and exhibited an active band at the corresponding position (Figure 17). Chi18D was purified with a minor, smaller protein band and showed an active band (Figure 17). The results revealed that Chi18C and Chi18D were successfully expressed and purified in active forms.

### 2.8. Properties of the Purified Chi18C and Chi18D

**Figure 18:**
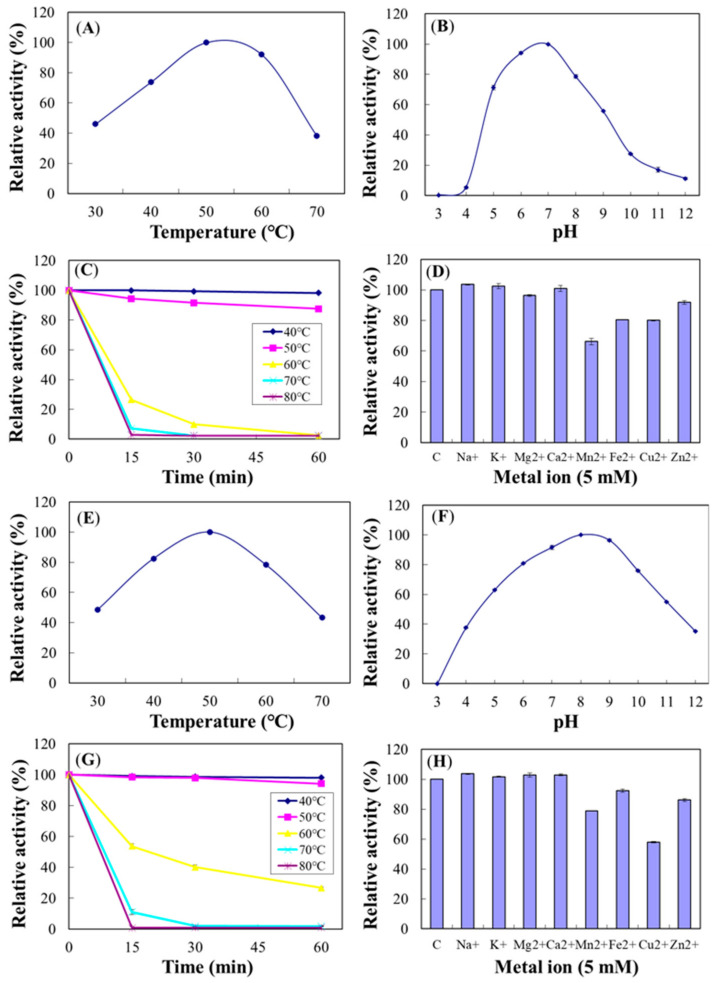
Characterization of Chi18C (**A**–**D**) and Chi18D (**E**–**H**). Effects of temperature (**A**,**E**), pH (**B**,**F**), thermostability (**C**,**G**), and metal ions (**D**,**H**) on purified Chi18C and Chi18D activities. The properties were characterized using the DNS method with colloidal chitin as a substrate.

The optimum temperatures for the purified Chi18C and Chi18D were identical at 50 °C, and the optimum pHs were 7.0 and 8.0, respectively (Figure 18). Both enzymes were stable at 50 °C for up to 1 h preincubation; however, Chi18C and Chi18D lost 73.6% and 46.3%, respectively, of their activity after 15 min preincubation at 60 °C (Figure 18). The half-lives of Chi18C and Chi18D at 60 °C were 10.2 and 19.1 min, respectively. For Chi18C, cations such as Mn^2+^, Cu^2+^, and Fe^2+^ decreased their activity to 66.2%, 80.1%, and 80.5%, respectively (Figure 18). In Chi18D, Cu^2+^ and Mn^2+^ decreased their activity to 57.9% and 78.9%, respectively (Figure 18).

### 2.9. Hydrolysis of Chitooligosaccharides by the Purified Chi18C and Chi18D

**Figure 19:**
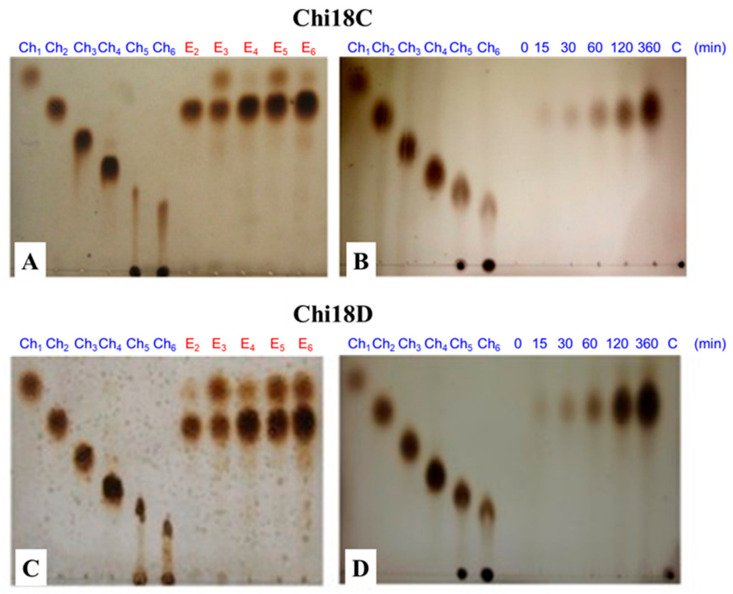
TLC profile of purified Chi18C (**A**,**B**) and Chi18D (**C**,**D**). TLC analysis of hydrolysis products of chitooligosaccharides (**A**,**C**) and colloidal chitin (**B**,**D**) as substrates for Chi18C and Chi18D. In (**A**,**C**): E_2_ to E_6_, *N*,*N*’-diacetylchitobiose (Ch_2_) through to hexa-*N*-acetylchitohexaose (Ch_6_) reacted with the enzyme. In (**B**,**D**): the reaction aliquots were sampled at the designated time above. The standards used were N-acetyl-D-glucosamine (Ch_1_), *N*,*N*′-diacetylchitobiose (Ch_2_), *N*,*N*′,*N*″-triacetylchitotriose (Ch_3_), tetra-*N*-acetylchitotetraose (Ch_4_), penta-*N*-acetylchitopentaose (Ch_5_), and hexa-N-acetylchitohexaose (Ch_6_).

When the purified Chi18C was reacted with the chitooligosaccharides (Ch_2_ to Ch_6_) as substrates, the major product of Ch_3_ to Ch_6_ was Ch_2_, whereas Ch_2_ was not hydrolyzed (Figure 19). The amount of Ch_2_ as product was proportional to the degree of polymerization of chitooligosaccharides; the hydrolysis of Ch_6_ was the highest among those tested. With colloidal chitin as a substrate, Ch_2_ was the main product, and the amount increased with the reaction time (Figure 19). When the purified Chi18D was reacted with the chitooligosaccharides and colloidal chitin as substrates, the hydrolysis results were the same as those for Chi18C (Figure 19), suggesting that Chi18C and Chi18D are endochitinases. The monosaccharide Ch_1_ spots were observed in the reactions of E_3_ to E_6_, and the spots in E_3_ and E_5_ were more intensive than those in E_2_, E_4_, and E_6_. This pattern suggests that Chi18C and Chi18D hydrolyze chitooligosaccharides consisting of more than two units of NAG by degradation of Ch_3_ and Ch_5_ into Ch_2_ and Ch_1_.

## 3. Discussion

Marine bacterial chitinases are considered sources of energy, eco-friendly agents, and industrial biocatalysts [11]. In this study, we identified two strains, SK10 and SK15, as *Aeromonas* sp., among three isolates from a marine source, i.e., a mudflat. In a previous report, SK16 was deposited as a new species of *C. suncheonensis* sp. nov. [18]. Furthermore, four chitinase genes were cloned from them: *chi*18A and *chi*19B from *Aeromonas* sp. SK10, *chi*18C from *Aeromonas* sp. SK15, and *chi*18D from *C. suncheonensis* sp. nov. Three GH 18 family members, i.e., Chi18A, Chi18C, and Chi18D, were endochitinases, and GH 19 family member Chi19B was found to be an exochitinase, based on an inability to hydrolyze colloidal chitin but an ability to hydrolyze MUCh_2_.

No information about the chitinase gene from *Chitinibacter* sp. has been reported in the literature; however, recently, a chitinase gene was isolated and cloned from *C. tainanensis*, and an encoded enzyme was characterized [19].

Chi18A and Chi18C have a high identity, with a score of 80.46%; however, there are some differences in properties; in optimum temperatures, i.e., 50 °C and 60 °C, respectively; and in ion effects, i.e., Chi18A was activated by Mg^2+^ and K^+^ with relative activities of 120% and 117%, respectively, but Chi18C was not activated. In addition, Chi18A was inhibited by Fe^2+^, but Chi18C was inhibited by Ca^2+^.

Comparing amino sequences with other characterized chitinases, Chi18A, Chi18C, and Chi18D had low identity (4–48%) to the family of 18 chitinases [24,25,26], and Ch19B also had low identity (10.4–31.0%) with the other family of 19 chitinases reported [27,28,29].

The molecular masses of bacterial chitinases vary between 20 and 90 kDa [30]. Molecular masses of family 18 chitinases ranged between 16.4 and 92.7 kDa [17,24,31,32] (Table 2). Chi18A (92.7 kDa) and Chi18C (91.6 kDa) had the highest molecular masses in the family 18 chitinases, for instance, ChiC (92.7 kDa) from *Pseudoalteromonas* sp. DL-6 [32], while Chi18D was, on average, 70.8 kDa. Molecular masses of family 19 chitinases ranged from 24.8 to 70.6 kDa [27,28,29], and most of them had molecular masses of 24.8–39.8 kDa [27,33,34] (Table 2). Chi19B had the highest molecular mass, i.e., 70.6 kDa, followed by Chi19 from *Vibrio proteolyticus* (60.1 kDa) [29].

The optimum temperatures for family 18 chitinases ranged from 30 °C to 60 °C [33,35,36,37]. Chi18A and Chi18D had optimum temperatures with average values (50 °C). Chi18C had the highest optimum temperature, for instance, ChiA from *Nocardiopsis prasine* OPC-131 [33] and ChiA *Bacillus licheniformis* DSM8785 [38] (60 °C and 50–60 °C, respectively) (Table 2). Chi19B was optimally active at 50 °C, which was the average value of family 19 chitinases (Table 2).

Most of the family 18 chitinases were optimally active at acidic pH [35,36,37], whereas only a few were optimally active at alkaline, i.e., ChiC from *Streptomyces sp.* DA11 [24] and ChiC from *Pseudoalteromonas* sp. DL-6 [32] (pH 8.0 and 9.0, respectively). It is suggested that Chi18A, Chi18C, and Chi18D in this study are alkaline chitinases with optimum pHs of 8.0, 7.0–8.0, and 9.0–10.0, respectively (Table 2). Conversely, most of the family 19 chitinases were optimally active under acidic conditions, below pH 7.0 [33,34,39]. However, interestingly, Chi19B was optimally active under alkaline conditions, i.e., pH 9.0, suggesting that Chi19B is an alkaline chitinase.

The ion effects on family 18 chitinases were diverse. Chi18A, Chi18C, and Chi18D were significantly inhibited by Cu^2+^, like most of the family 18 chitinases, except ChiC from *Streptomyces* sp. DA11 [24] and Sm4 from *Stenotrophomonas maltophilia* [40]. Chi18A and Chi18C were inhibited by Mn^2+^, whereas most of the family 18 chitinases were activated by Mn^2+^, such as ChiC [24], G22 from *Stenotrophomonas rhizophila* G22 [25], and SaChiA4 from *Streptomyces albolongus* ATCC 27,414 [15], which is similar to Chi18D (Table 2).

In general, endochitinase cleaves substrates of a large size by attacking the inside randomly, i.e., endo-acting, to produce oligomers and finally disaccharide *N*,*N*″-diacetylchitobiose as a main product. Exochitinase cleaves the substrates by hydrolyzing non-reducing ends to produce the disaccharide as a product but does not attack inside the substrates. The patterns can be distinguished experimentally by primarily observing halo formation abilities for colloidal chitins (large-sized substrates) and additionally disaccharide production for oligosaccharides (small-sized substrates) as a main product. If both are observed, the enzyme is an endochitinase. However, if a halo is not formed for colloidal chitins, the enzyme is an exochitinase. In this study, Chil18C and Chi18D could degrade colloidal chitin to form halos, as well as Chi18A, and the major product was dimer. Therefore, Chi18C and Chi18D, as well as Chi18A, showed endo-type reactions, i.e., endochitinase, like most of the family 18 chitinases [15,30,38], except ChiC [32] and BthChi74 from *Bacillus thuringiensis* [35], which showed an exo-type reaction, i.e., exochitinase (Table 2), producing Ch_2_ as its main products. Most of the family 19 chitinases were reported to be endochitinase. However, in our experiment, Chi19B exhibited an exo-type reaction based on non-hydrolytic results for colloidal chitin and hydrolytic results for MUCh_2_, which are similar to Ch_3_ in their structures. Like Chi19B, it was reported that Chi19 from *Vibrio proteolyticus* hydrolyzed colloidal chitin to release small soluble oligosaccharides at an early stage, noting that Chi19 was not a strict exo-type reaction and an exo-like chitinase because its products contained a small amount of Ch_3_ and Ch_4_ [29] (Table 2). Further study of the substrate specificity of Chi19B will be necessary.

**Figure 20:**
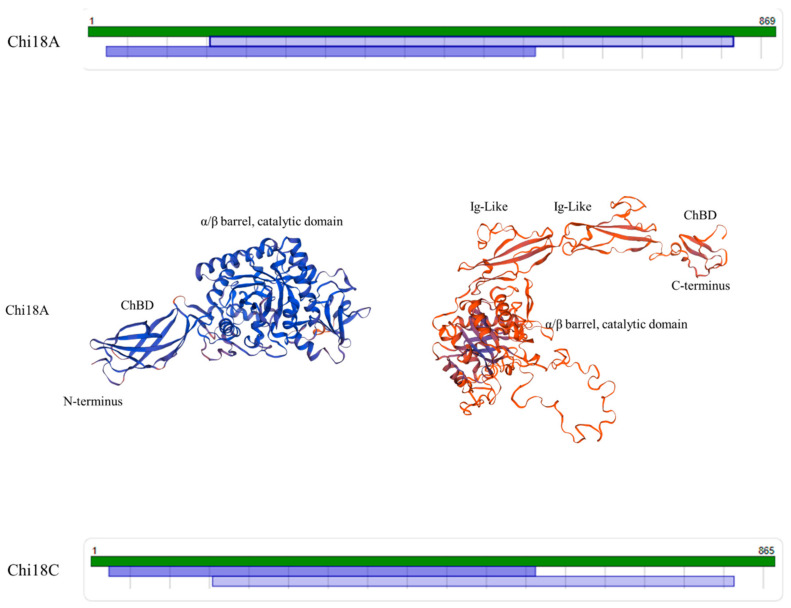

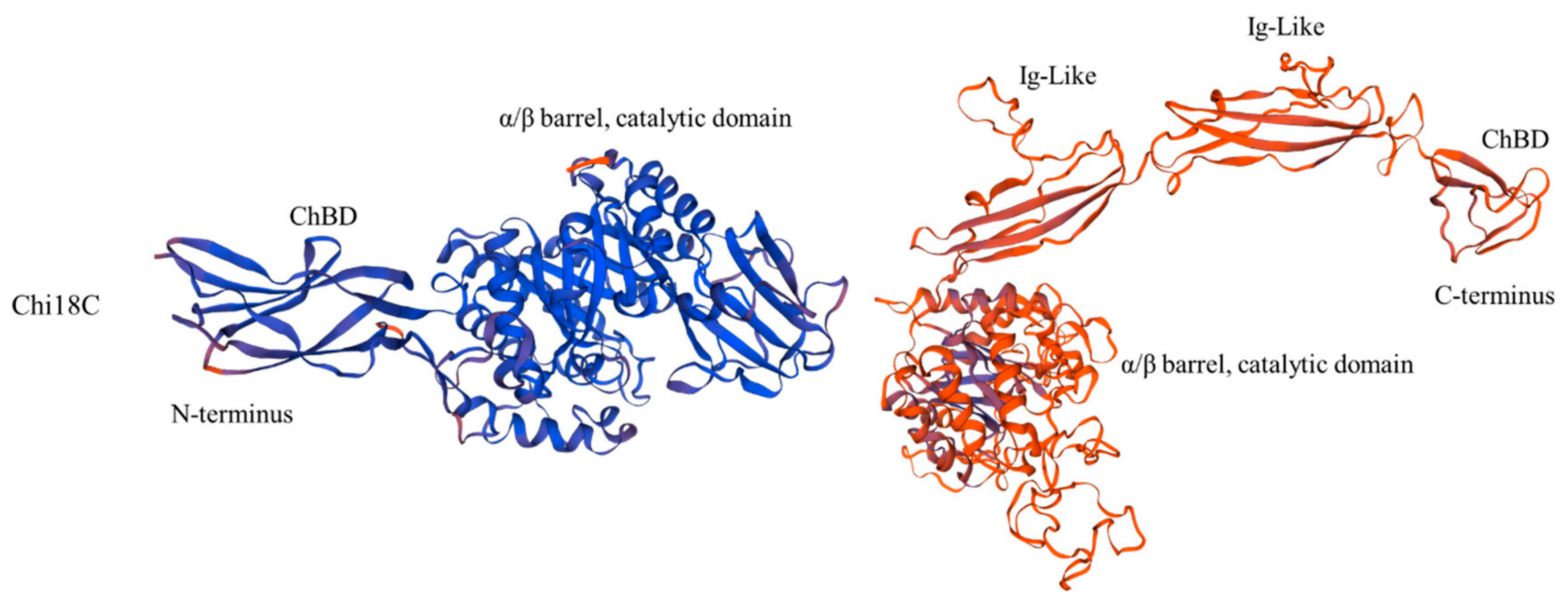
The predicted model map and 3D structures of Chi18A and Chi18C. For Chi18A, the N-terminal part- (24–565) and C-terminal part- (155–815) containing structures were predicted with the model templates of Chitinase A (PDB code: 1 x 6l.1.A; identity of 74.42%) and Chitinase60 (PDB code: 4mb3.1.A; identity of 18.40%), respectively. For Chi18C, the N-terminal part- (155–812) and C-terminal part- (24–562) containing structures were predicted with Chitinase A (PDB code: 1ffr.1.A; identity of 76.02%) and Chitinase60 (PDB code: 4hme.1.B; identity of 17.62%), respectively, like Chi18A.

In predicting 3D structures, models of Chi18A and Chi18C showed the highest identity to Chitinase A (PDB code: 1 x 6l.1.A) and Chitinase A (PDB code: 1ffr.1.A), respectively. Due to the high molecular weights of Chi18A (92.7 kDa) and Chi18C (91.6 kDa), which are unique cases of chitinases, there were no predicted models that covered full domains entirely. However, two structures were predicted, matching the N-and C-terminal parts of Chi18A or Chi18C with the highest identity (Figure 20). In the N-terminal part-containing structure of Chi18A (24–565), ChBD and the catalytic domain were predicted to be A24–A131 and L155–D565, respectively, with an identity of 74.42%. For the C-terminal part-containing structure of Chi18 A (155–815), two Ig-Like domains were predicted at P574–P758, which were previously reported [41] (Figure 20), and another ChBD was predicted at D768–Q809 with an identity of 18.40%. Chi18C showed similar patterns with Chi18A, i.e., N-terminal ChBD at A24–A108, catalytic domain at K155–Y562, Ig-like domains at P570–K752, and C-terminal ChBD at A768–Q809 (Figure 20). Collectively, Chi18A and Chi18C were predicted to have two ChBD and two Ig-like domains, suggesting that they promote substrate affinity with a large binding surface [41].

**Figure 21:**
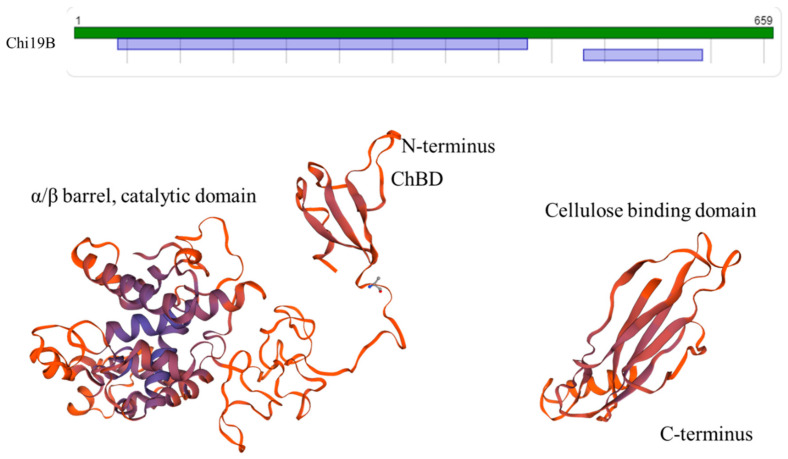
The predicted model map and 3D structure of Chi19B. The model templates of the N-terminal part- (42–427) and C-terminal part- (481–592) containing structures were chitinase C (PDB code: 1wvv 2.B, identity of 32.95%) and cellulose-binding protein (PDB code: 2yhg 1.A, identity of 22.73%).

Chi19B showed the highest identity (32.95%) with chitinase C (PDB code: 1wvv. 2.B). However, a model could not be predicted to cover the full domains (Figure 21). In the predicted model, ChBD and the catalytic domain were predicted at A24–G95 and K172–C427 in the N-terminal part- (42–427) containing structure, and the cellulose-binding domain was predicted at Y481–I592 in the C-terminal part- (481–592) containing structure (Figure 21). According to the 3D modeling result, we checked activity with carboxyl-methylcellulose (CMC) using an LB agar plate containing 1% CMC, and then stained with Congo red. However, activity was not detected.

**Figure 22:**
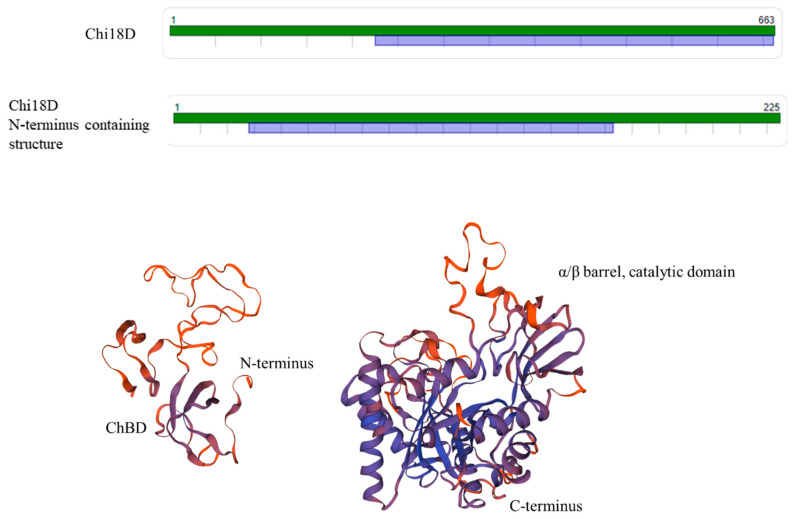
The predicted model map and 3D structure of Chi18D. The model templates of the N-terminal part- (31–161) and C-terminal part- (226–661) containing structures were deacetylase DA1 (PDB code: 4ny2.1.A; identity of 34.52%) and chitinase B (PDB code: 1kfw.1.A; identity of 44.01%), respectively.

Chi18D showed the highest identity (44.01%) with chitinase B (PDB code: 1kfw.1.A), but it could not cover the full domains. Therefore, we independently performed the N-terminal part-containing (1–225) structure and confirmed that it showed the highest identity (34.52%) with ChBD of Deacetylase DA1 (PDB code: 4ny2.1.A) (Figure 22). Collectively, the ChBD and catalytic domain were predicted at A31–Q161 and P226–A661, respectively (Figure 22).

These differences in their structures might suggest that they have different substrate specificities depending on the degree of polymerization of chitin, as observed in the halo formation differences for colloidal chitin hydrolysis.

## 4. Materials and Methods

### 4.1. Chemicals

Chitooligosaccharides, such as *N*,*N*′-diacetylchitobiose (Ch_2_), *N*,*N*′,*N*″-triacetylchitotriose (Ch_3_), tetra-*N*-acetylchitotetraose (Ch_4_), penta-*N*-acetylchitopentaose (Ch_5_), and hexa-*N*-acetylchitohexaose (Ch_6_)), were purchased from the Seikagaku Corporation (Tokyo, Japan). Isopropylthio-β-D-galactoside (IPTG) and 5-bromo4-chloro-3-indolyl-β-D-galactoside (X-gal) were purchased from Bioneer (Daejeon, Korea). *N*-acetyl-glucosamine (NAG or Ch_1_), 4-methylumbelliferyl-β-D-*N,N*′-diacetylchitobioside (MUCh_2_), chitin, dinitrosalicylic acid (DNS), and other chemicals were purchased from Sigma-Aldrich (St. Louis, MO, USA).

### 4.2. Isolation of Chitinolytic Bacterial Strains and Culture Conditions

Chitinolytic bacterial strains were previously isolated from an enriched mudflat in Suncheon Bay, Republic of Korea, using Luria–Bertani (LB) medium containing 0.2% colloidal chitin (LBCC) agar plates [18]. Colloidal chitin was prepared using Hsu and Lockwood’s [42] method with modifications. After incubation at 30 °C for three days, positive colonies with large zones of hydrolysis were selected. Extracellular chitinase activity in the supernatant was measured after being grown at 30 °C for 18 h in an LBCC medium.

### 4.3. Identification of Bacterial Strains

The genomic DNAs of the selected strains were isolated using a Genomic DNA Extraction Kit (SolGent, Daejeon, Korea), and their 16S rRNA sequences were determined by SolGent, as described previously [43]. Sequence similarities of the 16S rRNA and phylogenetic trees were searched and analyzed using the BLASTN program [44,45] on the NCBI website. The SK16 strain was previously deposited in KCTC and the DSM under the numbers KCTC 23839 and DSM 25421, respectively, as a new species, *C. suncheonensis* sp. nov., and its 16S rRNA sequence was deposited in GenBank under the accession number JN981166 [18]. Strains SK15 and SK10 were deposited in KCTC under the numbers KCTC 42713 and KCTC 42714, respectively, as *Aeromonas* sp. SK15 and *Aeromonas* sp. SK10. The 16S rRNA sequences of SK15 and SK10 were deposited in GenBank under the accession numbers MZ573230 and MZ573228, respectively.

### 4.4. Monitoring the Growth and Chitinolytic Activities of the Isolates SK10, SK15, and SK16

Three strains were cultured for 36 h at 37 °C with shaking at 150 rpm in 200 mL of LB medium 1-L flasks. Cell growth was monitored by sampling every 3 h. Effects of substrate addition on extracellular chitinolytic activity were analyzed for 36 h using culture supernatants sampled every 3 h with LB, LBCC, and 0.5 × LB containing 0.2% colloidal chitin.

### 4.5. Cloning and Analysis of Chitinase Genes from the Isolates SK10, SK15, and SK16

Chitinase genes were cloned from the isolates SK10, SK15, and SK16 using pUC19 and *Escherichia coli* DH5α (Yeastern Biotech. Co., Taipei, Taiwan), as described previously [43], with slight modifications. *E. coli* transformants were primarily grown on LB agar plates supplemented with ampicillin (50 μg ml^−1^), X-Gal, and IPTG for about 24 h at 37 °C. The transformants were tooth-picked to LBCC agar plates, grown for about 24 h at 37 °C, and the colonies with a hydrolysis zone were selected. The nucleotide sequence of the insert DNA was determined by SolGent. The conserved region of the gene was identified with BlastN or BlastP of BLAST of NCBI (http://www.ncbi.nlm.nih.gov, accessed on 18 August 2021), and a phylogenetic tree of the gene was constructed using DNA/MAN (Lynnon Biosoft, version 4.11, Quebec, QC, Canada). The signal peptide was predicted with SignalP 5.0 in CBS (http://www.cbs.dtu.dk/services/SignalP/, accessed on 18 August 2021) [46]. The molecular mass and pI of the encoded protein were predicted, and multiple alignments were constructed using DNA/MAN (Lynnon Biosoft, version 4.11, Quebec, QC, Canada). Identified chitinase gene sequences of *chi*18A, *chi*19B, *chi*18C, and *chi*18D were deposited in GenBank under the accession numbers MZ673655, MZ673656, MZ673657, and MZ673658, respectively. Three dimensional structures of them were predicted by SWISS-MODEL (https://swissmodel.expasy.org/, accessed on 21 August 2021).

### 4.6. Subcloning of Chitinase Genes from the Active Clone SK10-5

Three open reading frames (ORFs) in the active clone SK10-5 were amplified individually or combinatorially using the primer sets (Table 1). The insert DNA fragments of the six kinds of subclones were verified, and the activities of the subclones were analyzed on an LBCC agar plate.

### 4.7. Enzyme Assay

Chitinase activity was assayed in a 1.0 mL reaction mixture containing 50 mM sodium acetate buffer (pH 7.0) and 0.5% colloidal chitin. At the end of the reaction, for 30 min at 37 °C, the amounts of reducing sugar released were determined using the DNS method after centrifugation of the mixtures at 12,000 rpm for 5 min [47]. One unit of enzyme activity was defined as the amount of enzyme that liberated 1 μmol of reducing sugar per minute under the conditions. NAG was used as the standard for this method.

### 4.8. Biochemical Characterization of Chitinases

The effects of pH and temperature on enzyme activities were investigated using crude or purified enzymes at pH values with 50 mM of universal buffer (from pH 3.0 to 12.0) at temperatures from 40 to 80 °C and using 50 mM of sodium acetate buffer (pH 7.0). Enzyme thermostability was analyzed by preincubation without substrate for 0, 15, 30, and 60 min at 40–80 °C. The influence of various cations on enzyme activity was determined at concentrations of 5.0 mM for Na^+^, K^+^, Mg^2+^, Ca^2+^, Mn^2+^, Fe^2+^, Cu^2+^, or Zn^2+^.

### 4.9. Estimation of the Molecular Mass of the Chitinases by Activity Staining

The molecular masses of the chitinases were estimated using crude extracts employing the activity staining method, as previously described [48], except with MUCh_2_ instead of MUG, and with modification. Shortly, after SDS-PAGE, the gels were washed three times with 20% isopropanol for 15 min to remove SDS and with 50 mM of sodium acetate buffer (pH 7.0) three times for 15 min to renature the enzyme, then soaked in a buffer containing 2 mM of MUCh_2_ for 30 min at 4 °C. The gels were then transferred onto glass plates and incubated at 50 °C for 5–15 min. The MUCh_2_-hydrolyzing activity was photographed as fluorescent bands under UV light.

### 4.10. Site-Directed Mutagenesis

Three chitinase genes were changed using a site-directed mutagenesis method and a QuikChange II kit (Stratagene, Santa Clara, CA, USA). Primer sets were designed to mutate Glu (a tentative catalytic residue of each enzyme) to Ala (Table 3). The substitutions were made by PCR amplification using 160 ng DNA, 10 pmol primer, 2.5 mM dNTP, and 2.5 U of *Pfu* Ultra HF DNA polymerase (Stratagene) with pre-denaturation at 95 °C for 30 sec and 12 cycles (denaturation at 95 °C for 30 sec, annealing at 55 °C for 60 sec, and extension at 68 °C for 6 min). The PCR products were treated with *Dpn*I, and the resulting products were transformed to *E. coli* XL-1 blue super-competent cells (Stratagene). The substitutions were confirmed by nucleotide sequencing.

### 4.11. Expression and Purification of Two Chitinases from SK-15 and SK-16 Clones

Two chitinases of the active clones of SK-15 and SK-16 were highly expressed using a pET28a(+) vector. For pET expressions of Chi18C from SK-15 and Chi18D from SK16, primers were designed and used (Table 4).

Expression of the protein was monitored by varying the IPTG concentrations (0.5 and 1.0 mM) and induction times (6 and 12 h). After each clone was cultured for 12 h, suspended with the lysis buffer, incubated for 15 to 30 min on ice, and centrifuged at 12,000× *g* for 15 min at 4 °C under the native conditions, the supernatant was loaded into the Ni-NTA column (Qiagen, Hilden, Germany), washed, and eluted with the buffer following the manufacturer’s protocol. Protein concentrations were determined at 595 nm by the Bradford method using bovine serum albumin (BSA) as standard [49]. Sodium dodecyl sulfate–polyacrylamide gel electrophoresis (SDS-PAGE) was conducted on 11.5% polyacrylamide gels [50], which were stained with Coomassie Brilliant Blue R-250 or a Plus One Silver Staining Kit (GE Healthcare, Uppsala, Sweden).

### 4.12. Hydrolysis of Chitooligosaccharides and Colloidal Chitin by Purified Chitinases

The end products of the chitooligosaccharides were analyzed by incubating 0.1 U of the enzyme for 6 h and 12 h at 50 °C in the presence of 5 mM chitooligosaccharides, Ch2 to Ch6, or 0.2 U of the enzyme for 24 h with 0.5% colloidal chitin in 50 mM sodium acetate (pH 7.0). Reaction products were separated using thin-layer chromatography on a Silica gel 60 F254 plate (20 × 20 cm) (Merck, Darmstadt, Germany) with 1-butanol: acetic acid: water = 2:1:1 (*v*/*v*) as the developing solvent [51]. The products were visualized by spraying with ethanol–sulfuric acid (95:5, *v*/*v*) followed by drying for 10 min at 100 °C.

## 5. Conclusions

In this study, four chitinases were cloned and characterized from two *Aeromonas* spp. and *C. suncheonensis*. In an active clone from SK10, a gene cluster was found containing Chi18A, Pro2K, and Chi19B. It was found that Chi18A was an endochitinase and that Chi19B was an exochitinase based on the combinatorial amplification of the genes and hydrolysis experiments for colloidal chitin and MUCh_2_. Pro2K was a metalloprotease; however, its role was unclear in this study. Chi18C and Chi18D were cloned from SK15 and SK16, respectively. In the activity staining analysis, the molecular masses of Chi18A, Chi19B, Chi18C, and Chi18D were 96, 74, 95, and 73 kDa, respectively, corresponding to the expected sizes of the proteins.

Furthermore, Chi18C and Chi18D were purified after expression using a pET vector. Using the purified enzymes, it was found that both hydrolyzed chitooligosaccharides and colloidal chitin produce *N*,*N*′-diacetylchitobiose as the major product, indicating that both are endochitinases. In summary, the four chitinases in this study had signal peptides and, interestingly, had high molecular masses and alkaline preferences compared to other chitinases. In the 3D model prediction, Chi18A and Chi18C were predicted to have two ChBDs and two Ig-like domains, such that they would be expected to promote substrate affinity by a large binding surface. Chi18D was predicted to have a ChBD at the N-terminus. Chi19B was predicted to have a ChBD at the N-terminus and a cellulose-binding domain at the C-terminus; however, cellulolytic activity was not detected. In addition, Chi18A, Chi18C, and Chi18D were endochitinases with ChBD, and Chi19B was an exochitinase. Further studies on substrate specificity will be necessary, however. These four chitinases can be useful for breaking chitinous materials and producing small chitooligosaccharides.

## Figures and Tables

**Table 1:** Primers for amplification of individual or combinatorial ORFs of the three ORFs of clone SK10-5.

Primer Name		Primer Sequence	
ORF(1) F	5′	GAAACCAGGAATTCGTCCGAATTTTAATCG	3′
ORF(1) R	5′	TGGCTCTGGGCAAACTGG	3′
ORF(2) F	5′	CGGTGCAGGACAAGGTCT	3′
ORF(2) R	5′	AGCCCCTCCACCAGCTTGA	3′
ORF(3) F	5′	AGCAGATCACCGTCTCGC	3′
ORF(3) R	5′	TCAGCTGCGCTGCCAGTTCAGCATG	3′
ORF(1+2) F	5′	GAAACCAGGAATTCGTCCGAATTTTAATCG	3′
ORF(1+2) R	5′	AGCCCCTCCACCAGCTTGA	3′
ORF(2+3) F	5′	CGGTGCAGGACAAGGTCT	3′
ORF(2+3) R	5′	TCAGCTGCGCTGCCAGTTCAGCATG	3′
ORF(1+2+3) F	5′	GAAACCAGGAATTCGTCCGAATTTTAATCG	3′
ORF(1+2+3) R	5′	TCAGCTGCGCTGCCAGTTCAGCATG	3′

**Table 2:** Comparison of Chi18A, Chi19B, Chi18C, and Chi18D with other characterized chitinases.

Family 18																
Protein	Accession	Source	Homology (%) *			AA	MW (kDa)	Opt. Temp. (°C)	Opt. pH	Ion Effects			Major Product	ChBD **	Type of Reaction	Ref.
			A	C	D					Activating		Inhibiting				
Chi18A	MZ673655	*Aeromonas* sp. SK10		80	13	869	92.7	50	8.0	Mg^2+^, K^+^		Cu^2+^, Fe^3+^, Mn^2+^		O	Endo	This study
Chi18C	MZ673657	*Aeromonas* sp. SK15	80		12	865	91.6	60	7.0–8.0			Cu^2+^, Mn^2+^, Ca^2+^	Ch2	O	Endo	
Chi18D	MZ673658	*Chitinibacter suncheonensis* sp. nov. SK16	13	12		664	70.8	50	9.0–10.0	Mn^2+^		Cu^2+^	Ch2	O	Endo	
SaChiA4	AXY65110	*Streptomyces albolongus* ATCC 27414	9	4	4	427	44.7	50	5.0	Mn^2+^		Cu^2+^, Fe^3+^	Ch2		Endo	[15]
UMCda	QJX74482	*Myxococcus fulvus* UM01	3	4	6	234	26.1	35	7.0						Endo	[17]
ChiC	ABY83190	*Streptomyces* sp. DA11	6	4	4	148	16.4	50	8.0	Mn^2+^, Cu^2+^, Mg^2+^		Fe^2+^, Ba^2+^			Endo	[24]
G22	BBN21352	*Stenotrophomonas rhizophila* G22	12	12	32	702	72.6	37	5.9	Mn^2+^, Ca^2+^		Cu^2+^, Fe^2+^	Ch2	O	Endo	[25]
Chit62	ADR30609	*Serratia marcescens*	48	37	12	566	61.4	55	6.0							[26]
MUJ	AAB70917	*Stenotrophomonas maltophilia*	11	13	20	700	72.3	45	6.8	Ca^2+^, Mg^2+^		Hg^2+^, Cu^2+^, Zn^2+^		O		[31]
ChiC	AGU01018	*Pseudomonas* sp. DL-6	43	34	12	876	92.7	30	9.0	Na^+^, K^+^, Ca^2+^		Fe^3+^, Zn^2+^, Cu^2+^	Ch2	X	Exo	[32]
ChiA	BAC45251	*Nocardiopsis prasine* OPC-131	7	4	4	336	35.2	60	7					X	Endo	[33]
BthChi74	BAW98208	*Bacillus thuringiensis*	19	13	13	676	74.4	55	4.0–6.0				Ch2	O	Exo	[35]
rCHI-2	QIM58707	*Serratia marcescens*	11	9	9	499	55.6	55	6.0	Cr^2+^, K^+^, Pb^2+^		Ag^+^, Cu^2+^	Ch2		Endo	[36]
*Tf* Chi18A	Q47RL1	*Thermobifida fusca* YX	13	10	13	451	50.6	40–45	6.5				Ch2	O	Endo	[37]
ChiA	ACK44109	*Bacillus licheniformis* DSM8785	20	13	12	580	64.0	50–60	4.0–5.0				Ch2		Endo	[38]
Sm4	BAP19085	*Stenotrophomonas maltophilia*	12	13	21	701	73.0	45	5.6			Hg^2+^			Endo	[40]
**Family 19**																
**Protein**	**Accession**	**Source**	**Homology** (**%**)			**AA**	**MW** (**kDa**)	**Opt**. **Temp**. (°**C**)	**Opt. pH**		**Ion Effects**		**Major Product**	**ChBD** **	**Type of Reaction**	**Ref**.
											**Activating**	**Inhibiting**				
Chi19B	MZ673656	*Aeromonas* sp. SK10				659	70.6	50	9.0		Mn^2+^, Mg^2+^, K^+^	Zn^2+^, Cu^2+^, Fe^3+^		X	Exo	This study
BcChiA	BAF99002	*Bryum coronatum*	10.4			228	24.8							X	Endo	[27]
Chi19MK	BCJ03976	*Lysobacter* sp. MK9-1	11.3			311	33.7						Ch2	O		[28]
Chi19	BAE86996	*Vibrio proteolyticus*	31.0			544	60.1	40	5.5–7.0				Ch2	O	Exo-like	[29]
ChiB	BAC45252	*Nocardiopsis prasine* OPC-131	11.6			296	31.5	60	6.0					O		[33]
Chi35	BAA88833	*Streptomyces thermoviolaceus*	12.3			377	39.8	60	5–6					O		[34]
Chi25	BAA88834		11.1			269	28.7	70	5–6					X		
ChiIS	AAT27430	*Streptomyces sp. MG3*	11.4			303	31.8	50	5–7					O		[39]

* A, homology with Chi18A; C, homology with Chi18C; D, homology with Chi18D. ** O and X indicate the presence and absence of the chitin binding domain (ChBD), respectively.

**Table 3:** Primers for site-directed mutagenesis.

Primer Name		Primer Sequence		mer
Chi18A F	5′	ATATCGACTGGGCATTCCCGGGTGG	3′	25
Chi18A R	5′	CCACCCGGGAATGCCCAGTCGATAT	3′	25
Chi18C F	5′	ACATCGACTGGGCGTTCCCGGGCGGA	3′	26
Chi18C R	5′	TCCGCCCGGGAACGCCCAGTCGATGT	3′	26
Chi18D F	5′	GATATCGATTGGGCATTCCCAGGTGTT	3′	27
Chi18D R	5′	AACACCTGGGAATGCCCAATCGATATC	3′	27

**Table 4:** Primers for pET expressions of Chi18C and Chi18D.

Primer Name		Primer Sequence		mer
Chi18C F	5′	TTTGAATTCATGTTAAGTCCAAAACTTTCC	3′	30
Chi18C R	5′	TTTAAGCTTTCAGTTGCAGCTCGCC	3′	25
Chi18D F	5′	TTTGGATCCATGAGGATGGAGACCCTTATG	3′	30
Chi18D R	5′	TTTGAGCTCTTGACGAGCTTTACCCATTC	3′	29

## Data Availability

The data presented in this study are available on request from the corresponding author.

## Supplementary Materials

The following are available online at https://www.mdpi.com/article/10.3390/ijms222312822/s1.
