# Supplementary material for: Molecular Characterization of Four Alkaline Chitinases from Three Chitinolytic Bacteria Isolated from a Mudflat
Source: Int J Mol Sci. 2021 Nov 26;22(23):12822. doi: 10.3390/ijms222312822 (PMC8658002; doi:10.3390/ijms222312822)
Supplement: Supplementary file 1 [file ijms-22-12822-s001.zip › ijms-1448423-supplementary.pdf]

Supplementary Materials

Molecular Characterization of Four Alkaline Chitinases from Three Chitinolytic Bacteria Isolated from a Mudflat

Sung Kyum Kim [1], Jong Eun Park [2], Jong Min Oh [2] and Hoon Kim [2,*]

[1] Department of Agricultural Chemistry, Sunchon National University, Suncheon 57922, Korea; kimsk0851@scnu.ac.kr
[2] Department of pharmacy, and Research Institute of Life Pharmaceutical Sciences, Sunchon National University, Suncheon 57922, Korea; 1200113@s.scnu.ac.kr (J.E.P), ddazzo005@naver.com (J.M.O)
* Correspondence: hoon@scnu.ac.kr; Tel.: +82617503751

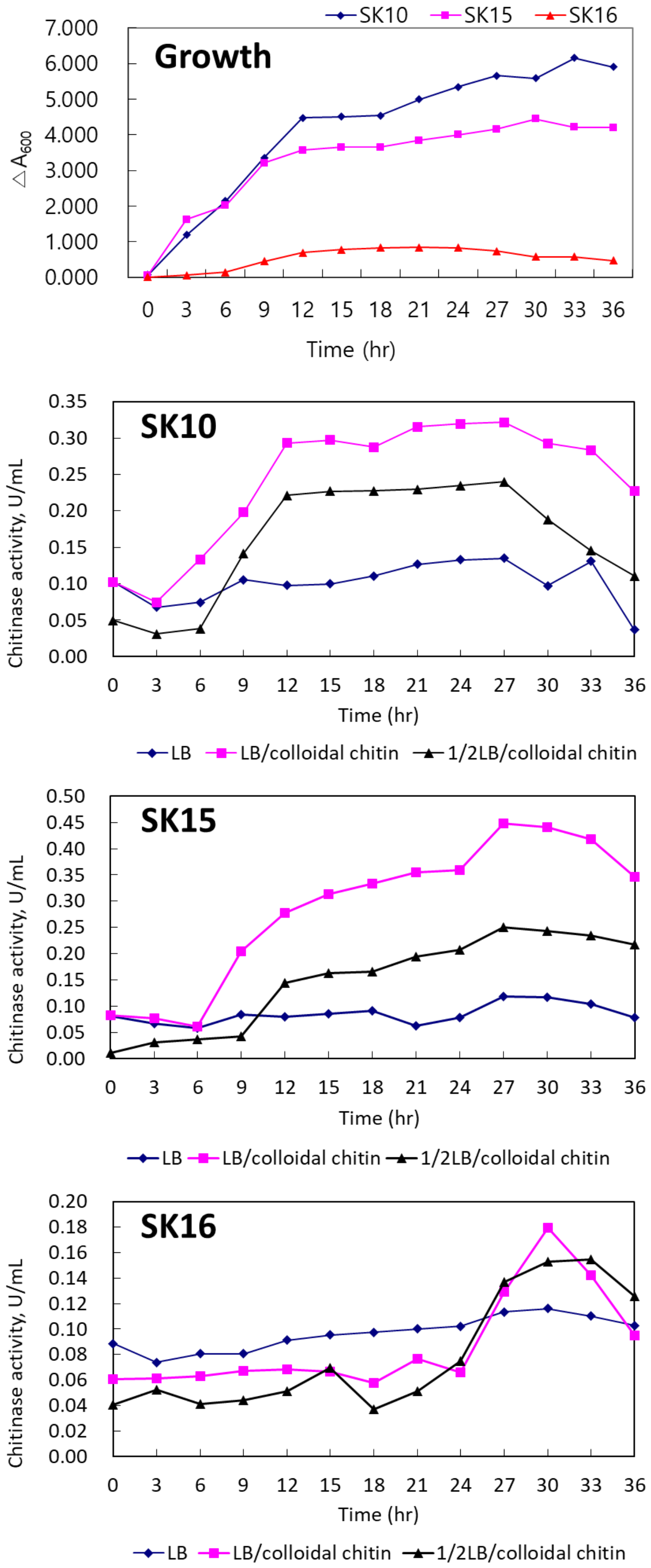

Figure S1. Growth of the isolates SK10, SK15, and SK16 in LB medium, and influence of colloidal chitin as the substrate on the chitinase production by the isolates. The isolates were cultured with shaking at 150 rpm at 30 °C. -◆-, LB; -■-, LB/colloidal chitin; -▲-, 0.5 × LB/colloidal chitin.

(A)

```
1       GACACAAAAAAGTGCCTTTTCTTCTCGTTTGAGGCTCATTTTCGATAAAAAGTGGTTTAT
61      TAATAACCGCTCGGGGTTTGCCCTCCCTCCTTTGACTCCCTTATAGTCAGGTCGCGATT
121     ATTTTTGTCGCAAATCAAGTTGTGCAAAAGGCAAACCGGATGAAAGTCCGGGACGCAAAG
181     CTTCCGGTCTAAGGGTCAGCAGTACCTAAGATAGCGGGGCCACCACAGGTTGTTTCGTCC
241     ATGGGCCAAGCCATGGTGCGATGTTGTCTGCGTTACCTGTCAGGTTGCGTTGCCTTTTTC
301     GTTATCTATTCCAAGAACGAAATAAGGATATTCAACTATGTTAAGTCCAAAACCCTCCGT
1                                                 M  L  S  P  K  P  S  V
361     ACTGGCGTTGTTGGTCGGGGGTCTGTGTGCCTCCTCCGCCTTCGCTGCCGCCCCCGGCAA
9         L  A  L  L  V  G  G  L  C  A  S  S  A  F  A  A  A  P  G  K
                       Signal peptide
421     ACCCACCATTGGCTGGGGCGAAACCAAGTTCGCCATCGTCGAAGTGGATCAAGCCGCCAC
29        P  T  I  G  W  G  E  T  K  F  A  I  V  E  V  D  Q  A  A  T
481     CTCCTACAACAAGCTGGTCAAGGTGCACGCCGATGGCGCGCCGGTCAGCGTGAGCTGGAA
49        S  Y  N  K  L  V  K  V  H  A  D  G  A  P  V  S  V  S  W  N
541     CCTCTGGTCCGGTGATGTCGGCCAGACCGCCAAGGTACTGCTCGACGGCAAGGAGGTGTG
69        L  W  S  G  D  V  G  Q  T  A  K  V  L  L  D  G  K  E  V  W
601     GTCCGGCGCCGCGTCCGCCTCCGGTACCGCCAACTTCAAGGTCACCAAGGGGGGCCGCTA
89        S  G  A  A  S  A  S  G  T  A  N  F  K  V  T  K  G  G  R  Y
661     CCAGATGCAGGTGGCGCTGTGCAACGCCGACGGCTGCACCCTCTCCGACAAGAAAGAGAT
109       Q  M  Q  V  A  L  C  N  A  D  G  C  T  L  S  D  K  K  E  I
721     CCTGGTGGCCGACACCGACGGCAGCCATCTGCTGCCGCTCAAGGCGCCGCTCAAGGAGAA
129       L  V  A  D  T  D  G  S  H  L  L  P  L  K  A  P  L  K  E  N
781     CAACAAGCCCTACGTCAACAAGACCGGCAAGGTGATGGGGGCCTACTACGTGGAGTGGGG
149       N  K  P  Y  V  N  K  T  G  K  V  M  G  A  Y  Y  V  E  W  G
841     GGTCTATGGTCGCAAGTTCACCGTGGACAAGATCCCGGCCCAGAACCTGACCCACATCCT
169       V  Y  G  R  K  F  T  V  D  K  I  P  A  Q  N  L  T  H  I  L
901     CTACGGCTTCACCCCGATCTGCGGTGGCAACGGCATCAACGACAGCCTGAAAGAGATCTC
189       Y  G  F  T  P  I  C  G  G  N  G  I  N  D  S  L  K  E  I  S
961     CGGCAGCTTCGAGGCGCTGCAGCGCTCCTGTGCCGGCCGCGAGGACTTCAAGGTCTCCAT
209       G  S  F  E  A  L  Q  R  S  C  A  G  R  E  D  F  K  V  S  I
1021    CCACGATCCCTGGGCTGCCATCCAGATGGGGCAGGGCAATCTCACCGCCTATGACGAACC
229       H  D  P  W  A  A  I  Q  M  G  Q  G  N  L  T  A  Y  D  E  P
1081    CTACAAGGGCAACTTCGGCAACCTGATGGCGCTCAAGAAGGCCTATCCGGACCTGAAGAT
249       Y  K  G  N  F  G  N  L  M  A  L  K  K  A  Y  P  D  L  K  I
1141    CCTCCCCTCCATCGGTGGCTGGACCCTCTCAGACCCCTTCTTCTTCTTCGGTGACAAGAC
269       L  P  S  I  G  G  W  T  L  S  D  P  F  F  F  F  G  D  K  T
1201    CAAGCGCGACACCTTCGTCGCCTCGGTGAAGGAGTATCTGCAAACCTGGAAATTCTTCGA
289       K  R  D  T  F  V  A  S  V  K  E  Y  L  Q  T  W  K  F  F  D
1261    CGGGGTGGATATCGACTGGGAATTCCCGGGTGGCCTCGGGGCCAACCCCAACCTCGGCAG
309       G  V  D  I  D  W  E  F  P  G  G  L  G  A  N  P  N  L  G  S
    Conserved region  Active site
1321    CCCCTCCGACGGCGAAACCTACGTGCTGCTGATGAAGGAGCTGCGCGCCATGCTCGACGA
329       P  S  D  G  E  T  Y  V  L  L  M  K  E  L  R  A  M  L  D  E
1381    GCTGAGCGCAGAGACCGGCCGCACCTACGAGCTCACCTCCGCCATCAGCGCCGGCGGTGA
349       L  S  A  E  T  G  R  T  Y  E  L  T  S  A  I  S  A  G  G  D
1441    CAAGATTGCCAAGGTGGACTATCGCGCCGCCCAGCAATACATGAATCACATCTTCCTGAT
369       K  I  A  K  V  D  Y  R  A  A  Q  Q  Y  M  N  H  I  F  L  M
1501    GAGCTACGACTTCAACGGCGCCTGGGCCAACACCGGGCTGGGCCACCAGACCGCGCTCTA
389       S  Y  D  F  N  G  A  W  A  N  T  G  L  G  H  Q  T  A  L  Y
1561    CGAGTCGAGCTGGGATCCCAACACCAAGTACACCACCGACAAGGGGGTCAAGGCGCTGCT
409       E  S  S  W  D  P  N  T  K  Y  T  T  D  K  G  V  K  A  L  L
1621    GGGCCAGGGCGTCGAGCCGGGCAAAGTGGTGGTGGGTGCCGCCATGTATGGTCGCGGCTG
429       G  Q  G  V  E  P  G  K  V  V  V  G  A  A  M  Y  G  R  G  W
1681    GACCGGCGTCAGCGGGTATCAGGGCAACAACCCCTTCACCGGCACCGCCACCGGCCCCAT
449       T  G  V  S  G  Y  Q  G  N  N  P  F  T  G  T  A  T  G  P  I
1741    TGCGGGAACCGAAGCGGAAGGGATCTGGGAGAAGGGGGTCATCGACTACCGTGGCATCGC
469       A  G  T  E  A  E  G  I  W  E  K  G  V  I  D  Y  R  G  I  A
1801    CAAGGGCAAGATGGCCGGCGACTGGCAGTACAGCTATGACGAAGTGGCCGAGGCACCGTC
489       K  G  K  M  A  G  D  W  Q  Y  S  Y  D  E  V  A  E  A  P  S
1861    CGTGTTCAAGGCTGCCACCGGCGAGCTCATCACCTTCGATGACGAGCGCTCGGTCAAGGC
509       V  F  K  A  A  T  G  E  L  I  T  F  D  D  E  R  S  V  K  A
1921    CAAGGGGCAGTACGTGCTGGCCAACCAGCTCGGCGGCATGTTCGCCTGGGAAATCGATGC
529       K  G  Q  Y  V  L  A  N  Q  L  G  G  M  F  A  W  E  I  D  A
1981    CGACAACGGCGACATTCTCAACGCCATGCACGAGGGGCTGGGCCACGGCGATAGCACTCC
549       D  N  G  D  I  L  N  A  M  H  E  G  L  G  H  G  D  S  T  P
2041    GCCGGTCAACAAGCCGCCGGTGGCCAACGCCGGCACCGATCTCGCGGTGACCGGGCCGGC
569       P  V  N  K  P  P  V  A  N  A  G  T  D  L  A  V  T  G  P  A
2101    CCAGCTGGTGCTGGATGGTTCCTCCTCCCGCGATCCGGAAGGAGCGGTGCTGAGCTACAG
589       Q  L  V  L  D  G  S  S  S  R  D  P  E  G  A  V  L  S  Y  S
2161    CTGGGCCCAGATCTCCGGTCCCAAGGCGACCCTGAGCGATGCCACCCAGGCCAAGGCCAA
609       W  A  Q  I  S  G  P  K  A  T  L  S  D  A  T  Q  A  K  A  K
2221    GGTGAGCGTCGATGCGGTGAGCCAGGACGTCAATCTGGTGTTCGAGCTGACCGTGACCGA
629       V  S  V  D  A  V  S  Q  D  V  N  L  V  F  E  L  T  V  T  D
2281    TGACCACAACCTCAGCGCCAAGGATCAGGTGGTGGTGACCAACAAGGCGCCGCAACCCAA
649       D  H  N  L  S  A  K  D  Q  V  V  V  T  N  K  A  P  Q  P  N
2341    CCTGCCGCCGGTCGTCACGGTGCAGGACAAGGTCTCGGTGCAGGCCGGCAAGCAGGTGAG
669       L  P  P  V  V  T  V  Q  D  K  V  S  V  Q  A  G  K  Q  V  S
2401    CATCACCGCCAGCGCCACCGACCCCAACGGCGACACCCTGAGCTATCAGTGGAGCGTGCC
689       I  T  A  S  A  T  D  P  N  G  D  T  L  S  Y  Q  W  S  V  P
2461    GGCCGGGGTTACCGCCAGCGGTCAGAACAGCAAGACCCTGGTGGTGACGGGCCCCAGCGT
709       A  G  V  T  A  S  G  Q  N  S  K  T  L  V  V  T  G  P  S  V
2521    CACCGTGGAGACCGGTTATGACCTCACCGTCACCGTGACCGACGGCGCCCTGGATGCCCA
729       T  V  E  T  G  Y  D  L  T  V  T  V  T  D  G  A  L  D  A  Q
2581    GGGCAAGACCCGCCTCACCGTGACCCCGGTCAGCGGTGGCGGCGACTGCAACGCCACCGA
749       G  K  T  R  L  T  V  T  P  V  S  G  G  G  D  C  N  A  T  D
2641    TCCGGATGCCGCCAACTGGCCGGCCTGGAAGGCCTCTACCACCTACAACGGTGGCGAGAA
769       P  D  A  A  N  W  P  A  W  K  A  S  T  T  Y  N  G  G  E  K
2701    GGTGAGCCACAACCAGCTGGTGTGGCAGGCCAAGTACTGGACCCAGGGCAATGAACCGAG
789       V  S  H  N  Q  L  V  W  Q  A  K  Y  W  T  Q  G  N  E  P  S
2761    CCGCACCGCCGATCAGTGGAAGCTGATAAGCCAGGTGCAGCTGGGCTGGGATGCCAGCGT
809       R  T  A  D  Q  W  K  L  I  S  Q  V  Q  L  G  W  D  A  S  V
2821    CGCCTACAACGCCGGTGACGTGACCACCCACAACGGTCGCCAGTGGAAGGCCCAGTACTG
829       A  Y  N  A  G  D  V  T  T  H  N  G  R  Q  W  K  A  Q  Y  W
2881    GACCCGCGGCAACGAGCCCGGCAAGCACGACGTCTGGCAGGACATCGGCGCCGCCAGCTG
849       T  R  G  N  E  P  G  K  H  D  V  W  Q  D  I  G  A  A  S  C
2941    CAAATAACCTCACCGTTAAACCCATCGGGGCATGCCAGGCATGCCCCCTTTTTTCCCCAT
869       K  *
3001    AACAACAAAGAGCACCACCATGACCACATGCACCACACGTCTTGCCTGCCTGATCGGCGC
3061    AGCCCTGGCAAGCGGCCCGCTGCTGGCCGCCGTTCAGCCACCCACCCCGCTGGTCTTTGA
3121    CGCCACCCGTCCGCAGAACGATCTGCAGGGGTCGCTGCTGGCCGGCGTCCAGTTTGCCCA
3181    GAGCCAGATCCTGCCCGCCCATCCTCGTGAGGGGGACAACCAGCCCCGCCTCACCGCCCT
3241    GCGCAAGAGTCTGCTGCTGGTGCGCCCGCAGCAGGCCG
```

(B)

```
Chi18A of SK10  MLSPKPSVLALLVGGLCASSAFAAAPGKPTIGWGETKFAIVEVDQAATSYNKLVKVHADG
BAE87051        MLSPKLSLLALLVGGLCTTSAFAAAPGKPTIGSGPTKFAIVEVNQAASAYNQLVTVHKDG
CAD32933        MRKFNKPLLALLIGSTLCSAAQAATPGKPTLAWGNTKFAIVEVGQAATAYNNLVKLKTAA
AAB97779        MRKFNKPLLALLIGSTLCSAAQAAAPGKPTIAWGNTKFAIVEVDQAATAYNNLVKVKNAA
CAA85291        MRKFNKPLLALLIGSTLCSAAQAAAPGKPTIAWGNTKFAIVEVDQAATAYNSLVKVKNAA
                *      .****.*   ...* **.*****.  * ******** ***..** ** ..

Chi18A of SK10  APVSVSWNLWSGDVGQTAKVLLDGKEVWSGAASA-SGTANFKVTKGGRYQMQVALCNADG
BAE87051        APVSVTWNLWSGDVGQTAKVLLDGKEVWSGPASA-AGTANFKVTKGGRYQMQVALCNADG
CAD32933        D-VSVSWNLWNGDTGTTAKVLLNGKEVWSGASTGASGTANFKVNKGGRYQMQVALCNAEG
AAB97779        D-VSVSWNLWNGDAGTTAKILLNGKEAWSGPSTGSSGTANFKVNKGGRYQMQVALCNADG
CAA85291        D-VSVSWNLWNGDTGTTAKVLLNGKEAWSGPSTGSSGTANFKVNKGGRYQMQVALCNADG
                  ***.**** ** * ***.** *** *** ..  .*******.**************.*

Chi18A of SK10  CTLSDKKEILVADTDGSHLLPLKAPLKENNKPYVNKTGKVMGAYYVEWGVYGRKFTVDKI
BAE87051        CTLSDKKELMVADTDGSHLAPLNAPLKENNKPYANKSGKVVGAYYVEWGVYGRKFTVDKI
CAD32933        CTASDATEIVVADTDGSHLAPLKEPLLEKNKPYKQDSGKVVGSYFVEWGVYNRNFTVDKI
AAB97779        CTASDATEIVVADTDGSHLAPLKEPLLEKNKPYKQNSGKVVGSYFVEWGVYGRNFTVDKI
CAA85291        CSASDATEIVVADTDGSHLAPLKEPLLEKNKPYKQNSGKVVGSYFVEWGVYGRNFTVDKI
                *. **  *..********* **  ** * **** . .***.*.*.******.* ******

Chi18A of SK10  PAQNLTHILYGFTPICGGNGINDSLKEISGSFEALQRSCAGREDFKVSIHDPWAAIQMGQ
BAE87051        PAQNLTHILYGFTPICGGNGINDSLKEISGSFEALQRSCAGREDFKVSIHDPWAAIQMSQ
CAD32933        PAQNLTHLLYGFIPVCGGDGINDSLKEIEGSFQALQRSCQGREDFKVSIHDPFAALQKGQ
AAB97779        PAQNLTHLLYGFIPICGGNGINDSLKEIEGSFQALQRSCQGREDFKVSIHDPFAALQKAQ
CAA85291        PAQNLTHLLYGFIPICGGNGINDSLKEIEGSFQALQRSCQGREDFKVSIHDPFAALQKAQ
                *******.**** *.*** ********* ***.****** ************ **.*  *

Chi18A of SK10  GNLTAYDEPYKGNFGNLMALKKAYPDLKILPSIGGWTLSDPFFFFGDKTKRDTFVASVKE
BAE87051        GNLSAWDEPYKGNFGNLMALKQAHPDLKILPSVGGWTLSDPFYFLGDKTKRDTFVASVKE
CAD32933        KGVTAWDDPYKGNFGQLMALKQARPDLKILPSIGGWTVSDPFFFMGDKEKRDRFVGSVKE
AAB97779        KGVTAWDDPYKGNFGQLMALKQARPDLKILPSIGGWTLSDPFFFMGDKVKRDRFVGSVKE
CAA85291        KGVTAWDDPYKGNFGQLMALKQAHPDLKILPSIGGWTLSDPFFFMGDKVKRDRFVGSVKE
                 ...* *.*******.*****.* ********.****.****.* *** *** ** ****

Chi18A of SK10  YLQTWKFFDGVDIDWEFPGGLGANPNLGSPSDGETYVLLMKELRAMLDELSAETGRTYEL
BAE87051        FLQTWKFFDGVDIDWEFPGGQGANPSLGGPNDGATYVVLMKELRAMLDELEAETGRQYEL
CAD32933        FLQTWKFFGGADIDWEFPGGQGANPKLGSAQDGATYVQLMKDLRAMLDQLSAETGRKYEL
AAB97779        FLQTWKFFDGVDIDWEFPGGKGANPNLGSPQDGETYVLLMKELRAMLDQLSAETGRKYEL
CAA85291        FLQTWKFFDGVDIDWEFPGGKGANPNLGSPQDGETYVLLMKELRAMLDQLSAETGRKYEL
                .******* * ********* **** **   ** *** ***.******.* ***** ***

Chi18A of SK10  TSAISAGGDKIAKVDYRAAQQYMNHIFLMSYDFNGAWANTGLGHQTALYESSWDPNTKYT
BAE87051        TSAISAGGDKIAKVDYQAAQQYMDHIFLMSYDFSGAFDLTNLAHQTNLFASSWDPATKYT
CAD32933        TSAISAGKDKIDKVDYNTAQNSMDHIFLMSYDFYGAFDLKNLGHQTPLKAPAWKPDTAYT
AAB97779        TSAISAGKDKIDKVAYNVAQNSMDHIFLMSYDFYGAFDLKNLGHQTALNAPAWKPDTAYT
CAA85291        TSAISAGKDKIDKVAYNVAQNSMDHIFLMSYDFYGAFDLKNLGHQTALNAPAWKPDTAYT
                ******* *** ** *  **. * ********* **    .* *** *   .* * * **
```

In the original figure, the motif FDGVDIDWE (FGGADIDWE in CAD32933) is highlighted in red.

Figure S2. Nucleotide sequence of *chi*18A gene and amino acid sequence of Chi18A deduced from the gene (A) and amino acid sequence alignments of Chi18A with other chitinases (B).

(A)

```
1        CTCTATCCGGCCCTACACGGCGCCTACGGTTTTGCCTATGGCGATGACGGCGAGCGGCTC
61       GGTAACAGTGACTGCCAGCTGCAGGTGGAAACCCGCGACGGCCTGCTGCGCTTCAAGCTG
121      GCCAACCACAGGCTGAGCGCCAGCGTGATGAACAAGTTCCATGTCAACGTGCCGAGCGCG
181      AGTGAACCGCGCAGCGCCAGCGTGCTGTGCCGCAACCAGTCACAGGCCGAGACCCAGCTT
241      GCCCCGGCCCCGGCCGGGCTCGGTTACACGGTCAACGGCATGCCTCTGGCGGCACGCTGA
301      GTGTTTTTCTTCTTCCTCCTCGCGCAATGCGAGGTGCCACTGGCGGGGGCCAAGCCCCGC
1                                    M  R  G  A  T  G  G  G  Q  A  P
361      CTTTCATCAAGCAAGAGGTAAATATGATGACAAGAAAGTCCTCTGTGGCCACCCTGCTGG
12       P  F  I  K  Q  E  V  N  M  M  T  R  K  S  S  V  A  T  L  L
                                Signal peptide
421      CCGGGCTCTGTCTGAGCGGTCAGCTGTGGGCCGCCAGCGCCTATGACATCAATCAGGGCT
32       A  G  L  C  L  S  G  Q  L  W  A  A  S  A  Y  D  I  N  Q  G
481      ATGGCGGCGGCAGCCAGGTCAGTTTCAACGGCCAGATCTACGAGGCCAAGTGGTATGCCA
52       Y  G  G  G  S  Q  V  S  F  N  G  Q  I  Y  E  A  K  W  Y  A
541      ATCCGGGCCAGAGCCCGGGCGTACAGGTCGCCAACGAGTGGGATTCTCCGTGGGCCCTGC
72       N  P  G  Q  S  P  G  V  Q  V  A  N  E  W  D  S  P  W  A  L
601      TGGGGGCGGGTGAAGCGACGCCACCGGGTGACGGGGAGGGCATAGCCCCACCCGCGCAGG
92       L  G  A  G  E  A  T  P  P  G  D  G  E  G  I  A  P  P  A  Q
661      GAGGCAGCGGCGCCGCTGACGGCAGCACCGTCTACCTGAGCCAGAGCGAGCTGGATGCCA
112      G  G  S  G  A  A  D  G  S  T  V  Y  L  S  Q  S  E  L  D  A
721      AGGAACATGCGCTCACCGACTTCCCGGCCATGACGGCGGTCAAGGCCTCCATCGCCACCC
132      K  E  H  A  L  T  D  F  P  A  M  T  A  V  K  A  S  I  A  T
781      GCGACAACGCCGTGGTCGAGGCGGTAGCCCCCGGCCTGCCGCTCAACCCCGCCAACGTCA
152      R  D  N  A  V  V  E  A  V  A  P  G  L  P  L  N  P  A  N  V
841      AGCTGGTGGAGGGGCTGATCAGCGAATCGCAGTTCAACTTCCTGTTCCCGCTGCGGGCGC
172      K  L  V  E  G  L  I  S  E  S  Q  F  N  F  L  F  P  L  R  A
901      CGGAGTACACCTATCGCGGCTTCCTACAGGCGGTGGCCAAGTTCCCGGCCTTCTGCGGTG
192      P  E  Y  T  Y  R  G  F  L  Q  A  V  A  K  F  P  A  F  C  G
961      ACTACAGCGACGGACGCAACGCCGAAGCCATCTGCCGCAAGTCGCTGGCCACCATGTTTG
212      D  Y  S  D  G  R  N  A  E  A  I  C  R  K  S  L  A  T  M  F
1021     CCCACTTCGCCCAGGAGACCGGCGGCCACGAGAGCTGGCGCCCCGAGCCCGAGTGGCGTC
232      A  H  F  A  Q  E  T  G  G  H  E  S  W  R  P  E  P  E  W  R
1081     AGGGGCTGGTGTGGGTACGGGAGATGGGCTGGACCGAGCAGATGCGCGGCGGCTACAACG
252      Q  G  L  V  W  V  R  E  M  G  W  T  E  Q  M  R  G  G  Y  N
1141     CCGAGTGCCGGCCGGATGTGTGGCAGGGTCAGCAGTGGCCGTGCGGCAAGTTCGACAACG
272      A  E  C  R  P  D  V  W  Q  G  Q  Q  W  P  C  G  K  F  D  N
1201     GCGAGTTCAAGAGCTACTTCGGTCGCGGCGCCAAGCAGCTGTCGTACAACTACAACTACG
292      G  E  F  K  S  Y  F  G  R  G  A  K  Q  L  S  Y  N  Y  N  Y
1261     GCCCCTTCTCCGAGGCCATGTTCGGTACTGTGCGTACCCTGCTGGACAACCCGGAGAAAG
312      G  P  F  S  E  A  M  F  G  T  V  R  T  L  L  D  N  P  E  K
1321     TGGCAGACACCTGGCTCAACCTGGCCAGCGCCGTGTTCTTCTTCGTCTATCCACAGCCGC
332      V  A  D  T  W  L  N  L  A  S  A  V  F  F  F  V  Y  P  Q  P
1381     CCAAGCCCTCCATGCTGCACGTGATCGACGGCACCTGGCAGCCCAACGCCCACGACCGGC
352      P  K  P  S  M  L  H  V  I  D  G  T  W  Q  P  N  A  H  D  R
1441     AGAACGGCCTGGTGCCGGGCTTCGGGGTGACCACCCAGATCATCAACGGCGGGGTGGAGT
372      Q  N  G  L  V  P  G  F  G  V  T  T  Q  I  I  N  G  G  V  E
1501     GCGGCGGTGCGGCCGAGCATGCCCAGTCCCAGAACCGGATCGCCTACTACCAGGCCCAGG
392      C  G  G  A  A  E  H  A  Q  S  Q  N  R  I  A  Y  Y  Q  A  Q
1561     CCCAGTATCTGGGGGTGCCGGTGCCTGCCGACGAGGTGCTCGGTTGCAAGAACATGAAGG
412      A  Q  Y  L  G  V  P  V  P  A  D  E  V  L  G  C  K  N  M  K
1621     TCTTTGACGAGGCCGGCGCCGGCGCCCTGCCCATCTACTGGGAGAACGACTGGAGCTATG
432      V  F  D  E  A  G  A  G  A  L  P  I  Y  W  E  N  D  W  S  Y
1681     TGCCGGGCAACCCCAACGGTGGCAAGAGCTACGCCTGCAAGCTGGTGGGCTACCAGACCC
452      V  P  G  N  P  N  G  G  K  S  Y  A  C  K  L  V  G  Y  Q  T
1741     GCCTCAGCGCCTTCAAGGAGCGTGACTACAGCCGCTGCGTGCAGCACTTCTTCCCCGAGG
472      R  L  S  A  F  K  E  R  D  Y  S  R  C  V  Q  H  F  F  P  E
1801     TGGTGATCGAGGACAACGGCGGTGGCACCAACCTGCCGCCGGTGGCCAGCATCAGCGGCC
492      V  V  I  E  D  N  G  G  G  T  N  L  P  P  V  A  S  I  S  G
1861     CGAGCGAGGCGCTGGGCGGCGCTACCGTGCTGCTCTCCGGCCAGGGCTCGAGCGATCCGG
512      P  S  E  A  L  G  G  A  T  V  L  L  S  G  Q  G  S  S  D  P
1921     AAGGCAAGCCCCTCAGCTACGCCTGGACCCTGCCTGCCGGGGCCAGCGCCTCGGATCTGA
532      E  G  K  P  L  S  Y  A  W  T  L  P  A  G  A  S  A  S  D  L
1981     CGCAGTCCACCCTGAGCGCCATCCTGCCGCCGACCCCGACCAGCGACACCCGCTACGGCT
552      T  Q  S  T  L  S  A  I  L  P  P  T  P  T  S  D  T  R  Y  G
2041     TCGTGCTCAAGGTGACCGACGACCAGGGGCTGAGCCGCAGCGCCACCCACGAGATCCTGA
572      F  V  L  K  V  T  D  D  Q  G  L  S  R  S  A  T  H  E  I  L
2101     TCAAGGGCAGTGGCGGTACCACGCCGCCGGATGGCAACCCGCCCTACAAGGAGGGCAACG
592      I  K  G  S  G  G  T  T  P  P  D  G  N  P  P  Y  K  E  G  N
2161     CTTACAAGGCCGGTGACAAGGTGAGCAACCTGGGCGGCAACTACCAGTGCAAGCCCCATC
612      A  Y  K  A  G  D  K  V  S  N  L  G  G  N  Y  Q  C  K  P  H
2221     CCTACACCGGCTGGTGTGCCGGCGCAGCCTGGGCCTATGAGCCCGGCAAGGGCACGGCCT
632      P  Y  T  G  W  C  A  G  A  A  W  A  Y  E  P  G  K  G  T  A
2281     GGAGCGATGCCTGGATCAAGCTCTAACGGCATATCCGCGCAATAAAAAAGGCCCGTCAGG
652      W  S  D  A  W  I  K  L  *
2341     GCCTTTTTTCATGGGGTGAGCGCTCACAGCGGCTTTTGCCACAGGCTCAGCAGCAGATCC
2401     TGCTCAAGTTCGTCCGGTCCCTGATGGCTGAAGGCGTGGCGCTGCTCACCGCTGGCGCGC
2461     CAGCCGAGGCGGCTGGTCTCCAGGATGAAGCTGCGCAGCCCTTCCTCCGCAGGGCAGTA
```

(B)

```
chi19B of SK10  ----QSELDAKEHALTDFPAMTAVKASIATRDNAVVEAVAPGLPLNPANVKLVEGLISES
YP_855523       ----QSELDAKEHALTDFPAMTAVKASIATRDNAVVEAVAPGLPLNPANVKRVEGLLSEA
YP_204442       YKVNQSALDAKEAELTNFDVMNDVKASIATLDNESVERIIPGAESNPENVKRIEQIVSES
YP_001143050    ----QSELDAREQALTDFPAMAAVKASIATRDNAVVEAVAPGLALNPANVKRVEGLLSES
ZP_02347102     ----ASDLAAKEKALTDFPLMEAVKSSIQTLDNSAVEQIEPGRAANPANVKRVESILKEA
                     * * *.*  ** *  *  **.** * **  ** . **   ** *** .* .. *.

chi19B of SK10  QFNFLFPLRAPEYTYRGFLQAVAKFPAFCGDYSDGRNAEAICRKSLATMFAHFAQETGGH
YP_855523       QFNFLFPLRAPEYTYRGFLQAVAKFPAFCGDYSDGRNAEAICRKSLATMFAHFAQETGGH
YP_204442       TWNFLFPKRAPEYTYRHFLQATGKFPAFCGTYEDGRDSDAICKKSLATMFAHFTQETGGH
YP_001143050    QFNFLFPLRAPEYTYRGFLQAVAKFPAFCGDYSDGRNAEAICRKSLATMFAHFAQETGGH
ZP_02347102     DWDYLFPMRAPEYTYSNFLKAIGKFPAVCGTYTDGRDSDAICRKTLATMFAHFAQETGGH
                   .*** *******  **.*  **** ** * *** ..***.*.********.******

chi19B of SK10  ESWRPEPEWRQGLVWVREMGWTEQMRGGYNAECRPDVWQGQQWPCGKFDNGEFKSYFGRG
YP_855523       ESWRPEPEWRQGLVWVREMGWTEQMRGGYNAECRPDVWQGQQWPCGKFDNGEFKSYFGRG
YP_204442       TAHWEVPEWRQGLVHVREMGWTEEMRGGYNGECNPDVWQGKTWPCGTFDNGEFKSYFGRG
YP_001143050    ESWRPEPEWRQGLVWVREMGWTEQMRGGYNAECRPDVWQGQQWPCGKFENGEFKSYFGRG
ZP_02347102     ESWRDIPEWRQALVYLREVGWTEGQKGGYNGECNPDVWQGQTWPCGKDKDGDFLSYFGRG
                 .    ***** **  .**.****  .**** ** ******. ****    *.* ******

chi19B of SK10  AKQLSYNYNYGPFSEAMFGTVRTLLDNPEKVADTWLNLASAVFFFVYPQPPKPSMLHVID
YP_855523       AKQLSYNYNYGPFSEAMFGTVRTLLDNPEKVADTWLNLASAVFFFVYPQPPKPSMLHVID
YP_204442       AKQLSYNYNYGPFSDAMFGTVRTLLDNPEMVADTWLNLSSAVFFFVYPQPPKPSMLHVID
YP_001143050    AKQLSYNYNYGPFSEAMFGTVRTLLDNPEKVADTWLNLASAVFFFVYPQPPKPSMLHVID
ZP_02347102     AKQLSYNYNYGPFSDAMYGDVRPLLDKPELVADTWMNLASAVFFFVYPQPPKPSMLHVID
                **************.**.* ** *** ** *****.**.*********************

chi19B of SK10  GTWQPNAHDRQNGLVPGFGVTTQIINGGVECGGAAEHAQSQNRIAYYQAQAQYLGVPVPA
YP_855523       GTWQPNAHDRQNGLVPGFGVTTQIINGGVECGGAAEHAQSQNRITYYQAQAQYLGVPVPA
YP_204442       GTWQPNQADLNNGLVPGFGVTTQIINGGVECGGSIEVAQSLNRIKYYKEMANYLGVPIED
YP_001143050    GTWQPNDYDRQNGLVPGFGVTTQIINGGVECGGAAEHAQSQNRITYYQAQAQYLGVPVPA
ZP_02347102     GTWQPNDRDKANGLVSGFGVTIQIINGGVECGGADENAQSLNRIAYYKEFANYLKVPVPA
                ******  *  **** ***** ***********. * *** *** **.  *.** **.

chi19B of SK10  DEVLGCKNMKVFDEAGAGALPIYWENDWSYVPGNPNGGKSYACKLVGYQTRLSAFKERDY
YP_855523       DEVLGCKNMKYFDEAGAGALPIYWENDWSYVPGNPNGGKSYACKLVGYQTRFSAFKERDY
YP_204442       TEILGCKGMKQFDAAGAGAVNIYWEQDWGWSSETPD-GKAYACQLVGYQTPHSAFKFRDY
YP_001143050    DEVLGCKSMKYFDEAGAGALPIYWENDWSYVPGNPNGGKSYACKLVGYQTRFSAFKARDY
ZP_02347102     DEVLGCKKMKQFDEGGAGALPIYWEQDWGWSADTAD-GKTYSCQLVGYQTPYTAFKEGDY
                 *.**** ** **  ****. ****.**     .   **.*.*.******  .***  **
```

Figure S3. Nucleotide sequence of *chi*19B gene and amino acid sequence of Chi19B deduced from the gene (A) and amino acid sequence alignments of Chi19B with other chitinases (B).

(A)

```
1        ATGATGGCGTCTCTTTCGTTGTTATTTTCAGGTTTTGGTGAAACCTGAGTCCCTTTGGGT
61       GTCACTTTTGGCAGGTGGCACCCGGGCGACTCTCCTCTATAGTCATTCGGCGATTATTTT
121      TGTCGCAAATCAAGTTGTGCAAAAAGGCAAACCGGATGAAAGTCCGGGACGCAAAGCTTC
181      CGGTCTAAGGGTCTGCGTACCTAGGATAGCGGGGCCACCACAGGTTGTATCGTCCATGGG
241      CCAAGCCATGGTTGCGATGTTGTCTGCGTTACCTGGCAGGTTGCGTTGCCTTTTTCGTTG
301      TTAATTCCAATAACGAAATAAGGAAGTTCAAATATGTTAAGTCCAAAACTTTCCCTGCTG
1                                           M  L  S  P  K  L  S  L  L
361      GCGCTTCTGGTCGGGGGGCTTTGCACTACCTCCGCCTTCGCCGCTGCCCCGGGCAAACCC
10        A  L  L  V  G  G  L  C  T  T  S  A  F  A  A  A  P  G  K  P
                   Signal peptide
421      ACCATTGGATCCGGCCCCACCAAGTTTGCCATCGTTGAAGTCAATCAGGCCGCTTCGGCC
30        T  I  G  S  G  P  T  K  F  A  I  V  E  V  N  Q  A  A  S  A
481      TACAACCAGCTGGTGACCGTCCACAAGGATGGCGCTCCCGTGAGCGTGACCTGGAACCTC
50        Y  N  Q  L  V  T  V  H  K  D  G  A  P  V  S  V  T  W  N  L
541      TGGTCCGGTGACGTCGGACAGACCGCCAAGGTACTGCTCGATGGCAAGGAGGTGTGGTCA
70        W  S  G  D  V  G  Q  T  A  K  V  L  L  D  G  K  E  V  W  S
601      GGCCCGGCCTCCGCCGCGGGTACCGCCAACTTCAAGGTCACCAAGGGGGGGCGTTACCAG
90        G  P  A  S  A  A  G  T  A  N  F  K  V  T  K  G  G  R  Y  Q
661      ATGCAGGTGGCCCTGTGCAACGCCGACGGCTGCACCCTCTCCGACAAGAAGGAGCTGATG
110       M  Q  V  A  L  C  N  A  D  G  C  T  L  S  D  K  K  E  L  M
721      GTCGCCGATACCGACGGCAGCCACCTGGCGCCGCTCAATGCGCCCCTCAAGGAGAACAAC
130       V  A  D  T  D  G  S  H  L  A  P  L  N  A  P  L  K  E  N  N
781      AAGCCCTACGCCAACAAGTCCGGCAAGGTGGTGGGGGCCTACTACGTGGAGTGGGGTGTC
150       K  P  Y  A  N  K  S  G  K  V  V  G  A  Y  Y  V  E  W  G  V
841      TATGGTCGCAAGTTCACCGTGGACAAGATCCCGGCCCAGAACCTGACCCATATTCTCTAC
170       Y  G  R  K  F  T  V  D  K  I  P  A  Q  N  L  T  H  I  L  Y
901      GGCTTCACCCCCATCTGCGGTGGCAACGGCATCAACGACAGCCTCAAAGAGATCTCCGGC
190       G  F  T  P  I  C  G  G  N  G  I  N  D  S  L  K  E  I  S  G
961      AGCTTCGAGGCGTTGCAGCGCTCCTGTGCGGGCCGCGAAGACTTCAAGGTCTCCATCCAT
210       S  F  E  A  L  Q  R  S  C  A  G  R  E  D  F  K  V  S  I  H
1021     GATCCCTGGGCGGCGATCCAGATGTCCCAAGGCAACCTCAGCGCCTGGGATGAGCCCTAC
230       D  P  W  A  A  I  Q  M  S  Q  G  N  L  S  A  W  D  E  P  Y
1081     AAGGGCAACTTCGGCAACCTGATGGCCCTCAAACAGGCCCATCCGGACCTCAAGATTCTG
250       K  G  N  F  G  N  L  M  A  L  K  Q  A  H  P  D  L  K  I  L
1141     CCGTCTGTCGGCGGATGGACCCTCTCCGATCCCTTCTATTTCCTGGGTGACAAGACCAAG
270       P  S  V  G  G  W  T  L  S  D  P  F  Y  F  L  G  D  K  T  K
1201     CGCGATACCTTCGTCGCCTCGGTCAAAGAGTTCCTGCAGACCTGGAAATTCTTCGACGGC
290       R  D  T  F  V  A  S  V  K  E  F  L  Q  T  W  K  F  F  D  G
1261     GTGGACATCGACTGGGAGTTCCCGGGCGGACAGGGGGCCAACCCCAGCCTGGGCGGCCCG
310       V  D  I  D  W  E  F  P  G  G  Q  G  A  N  P  S  L  G  G  P
     Conserved region  Active site
1321     AATGACGGCGCCACTTATGTGGTGCTGATGAAGGAGTTGCGAGCCATGCTCGATGAACTG
330       N  D  G  A  T  Y  V  V  L  M  K  E  L  R  A  M  L  D  E  L
1381     GAAGCCGAGACCGGCCGCCAGTATGAGCTGACCTCGGCCATCAGCGCCGGCGGTGACAAG
350       E  A  E  T  G  R  Q  Y  E  L  T  S  A  I  S  A  G  G  D  K
1441     ATTGCCAAGGTGGATTATCAGGCGGCCCAGCAGTACATGAACCACATCTTCCTGATGAGC
370       I  A  K  V  D  Y  Q  A  A  Q  Q  Y  M  N  H  I  F  L  M  S
1501     TACGACTTCAGCGGCGCCTTCGACCTGACGAATCTGGCCCACCAGACCAACCTCTTTGCC
390       Y  D  F  S  G  A  F  D  L  T  N  L  A  H  Q  T  N  L  F  A
1561     TCCAGCTGGGATCCGGCCACCAAGTACACCACCGACAAGGGCGTCAAGGCGCTGCTGGGT
410       S  S  W  D  P  A  T  K  Y  T  T  D  K  G  V  K  A  L  L  G
1621     CAGGGGGTGACACCGGGCAAGATTGTGGTGGGGGCGGCCATGTATGGCCGTGGCTGGACC
430       Q  G  V  T  P  G  K  I  V  V  G  A  A  M  Y  G  R  G  W  T
1681     GGAGTGAAGAACTACCAGGCCGGCAACCCCTTCACCGGCACCGCTACCGGACCGGTGAGC
450       G  V  K  N  Y  Q  A  G  N  P  F  T  G  T  A  T  G  P  V  S
1741     GGTACCTGGGAAAATGGCGTAGTGGATTACCGCGACATCGTCAACAACCGCATGGGCGCA
470       G  T  W  E  N  G  V  V  D  Y  R  D  I  V  N  N  R  M  G  A
1801     GGCTGGGAGCAGGGCTATGACGAGTCGGCCGAGGCCCCCTATGTCTTCAAGGCCAGCAGT
490       G  W  E  Q  G  Y  D  E  S  A  E  A  P  Y  V  F  K  A  S  S
1861     GGCGACCTCATCACCTTTGACAACGACCGCTCGGTCAAGGCCAAAGGGCAGTACGTGCTG
510       G  D  L  I  T  F  D  N  D  R  S  V  K  A  K  G  Q  Y  V  L
1921     GCGAACCAGCTCGGCGGTCTGTTCGCCTGGGAAATTGATGCGGACAACGGCGACATCCTC
530       A  N  Q  L  G  G  L  F  A  W  E  I  D  A  D  N  G  D  I  L
1981     AACGCCATGCACGAGGGGCTGGGCCACGGCGAGGGTACGCTGCCGCCGGTCAACAAGCCG
550       N  A  M  H  E  G  L  G  H  G  E  G  T  L  P  P  V  N  K  P
2041     CCGGTTGCCAATGCCGGTAGCGATCTGAGTGCCACCGGCCCGGCCGAGGTGACCCTCAAC
570       P  V  A  N  A  G  S  D  L  S  A  T  G  P  A  E  V  T  L  N
2101     GGCAGCGCCTCCCACGATCCGGAAAACGGGGCGCTGACCTACAGCTGGAAACAGGTCTCC
590       G  S  A  S  H  D  P  E  N  G  A  L  T  Y  S  W  K  Q  V  S
2161     GGACCCCAGGCCAGCCTGCTGGATGCCACCCAGGCCAAAGCCCGTGCGGTACTGGATGCC
610       G  P  Q  A  S  L  L  D  A  T  Q  A  K  A  R  A  V  L  D  A
2221     GTCAGCAGCGACATCAATCTGGTGTTCGAGCTGACCGTGACCGACGATCAGGGGCTCTCG
630       V  S  S  D  I  N  L  V  F  E  L  T  V  T  D  D  Q  G  L  S
2281     GCCAAGGATCAGGTGGTGGTCACCAACAAGGCGCCGCAGCCGAACCTGCCTCCCGTGGTC
650       A  K  D  Q  V  V  V  T  N  K  A  P  Q  P  N  L  P  P  V  V
2341     AGCGTACCGGCCAGTGCGACCGTCGAGGCCGGCAAGCAGGTGAGCATCAAGGCCACCGCT
670       S  V  P  A  S  A  T  V  E  A  G  K  Q  V  S  I  K  A  T  A
2401     TCCGACCCCAATGGTGATGCCTTGAGCTATCAGTGGACAGTGCCGGCCGGGCTCAGCGCC
690       S  D  P  N  G  D  A  L  S  Y  Q  W  T  V  P  A  G  L  S  A
2461     ACCGGTCTGGACAGCGCGACCCTGGTAGTAACGGGCTCGAACGTGACCAGTGACACGGCC
710       T  G  L  D  S  A  T  L  V  V  T  G  S  N  V  T  S  D  T  A
2521     TACGATCTGACCCTGGTGGTCACCGATGGGGCGCTGGATGCCACAGCCGTTACCCGCCTG
730       Y  D  L  T  L  V  V  T  D  G  A  L  D  A  T  A  V  T  R  L
2581     ACCGTCAAGCCGGCCAGTACAGGTGGTGGCTGCGAGGCCAGCGATCCGGATGCGGCCAAC
750       T  V  K  P  A  S  T  G  G  G  C  E  A  S  D  P  D  A  A  N
2641     CACCCGGCCTGGAGTGCAGGTACCGTCTACAACACCAATGACAAGGTGAGCCACAAGCAA
770       H  P  A  W  S  A  G  T  V  Y  N  T  N  D  K  V  S  H  K  Q
2701     CTGGTGTGGCAGGCCAAGTATTGGACCCAGGGCAACGAGCCGAGCCGCACCGCCGATCAG
790       L  V  W  Q  A  K  Y  W  T  Q  G  N  E  P  S  R  T  A  D  Q
2761     TGGAAACTGGTGAGCCAGGTACAACTGGGTTGGGATGCCGGAGTGGTCTACAACGGCGGT
810       W  K  L  V  S  Q  V  Q  L  G  W  D  A  G  V  V  Y  N  G  G
2821     GATGTCACCAGCCACAACGGCCGCAAGTGGAAGGCCCAGTACTGGACCAAGGGCGATGAG
830       D  V  T  S  H  N  G  R  K  W  K  A  Q  Y  W  T  K  G  D  E
2881     CCCGGCAAGGCCGCCGTCTGGGTCGATCAGGGCGCGGCGAGCTGCAACTGATCTGCCGTG
850       P  G  K  A  A  V  W  V  D  Q  G  A  A  S  C  N  *
2941     ATGAGAAATGGGGCCGAGGGCCCCATTTTTGTCTTGCTGCGTGGTTGGGTGTCTCTTCCC
3001     TGTCAGGGAAAAGGCGCACTCGCTTTGTCAAAAAGGCCCGTCAGGGCCTTTTTTCATGGT
3061     ATATCACCGGTTCACAGTGGTTTTTGCCACAGGCTGAGCAGCAGATCCTCTAG
```

(B)

```
chi18C of SK15  MLSPKLSLLALLVGGLCTT----SAFAAAPGKPTIGSGPTKFAIVEVNQAASAYNQLVTV
CAD58828        MLSPKLSLLALLVGGLCTT----SAFAAAPGKPTIGSGPTKFAIVEVNQAASAYNQLVTV
ZP_01842476     MFKTQISTSALLVASALASTVSVSAFAAAPGKPTIGWGETKFAIIEVNQAATAYNQLVTI
ACE78180        ----MRKFNKPLLALLIGSTLCSAAQAAAPGKPTIAWGNTKFAIVEVDQAATAYNNLVKV
BAE87051        MLSPKLSLLALLVGGLCTT----SAFAAAPGKPTIGSGPTKFAIVEVNQAASAYNQLVTV
                           *.     .    .* *********  * *****.** ***.***.** .

chi18C of SK15  HKDGAPVSVTWNLWSGDVGQTAKVLLDGKEVWSGPASA-AGTANFKVTKGGRYQMQVALC
CAD58828        HKDGAPVSVTWNLWSGDVGQTAKVLLDGKEVWSGPASA-AGTANFKVTKGGRYQMQVALC
ZP_01842476     -KDAADVSVSWNLWSGDVGNKAQVLLNGNVAWEGPSGA-SGTANFKVNKGGRYQMQVQLC
ACE78180        -KNAADVSVSWNLWNGDTGTTAKVLLNGKEAWSGPSTGSSGTANFKVNKGGRYQMQVALC
BAE87051        HKDGAPVSVTWNLWSGDVGQTAKVLLDGKEVWSGPASA-AGTANFKVTKGGRYQMQVALC
                 *  * ***.**** ** *  *.*** *   * **.   .*******.********* **

chi18C of SK15  NADGCTLSDKKELMVADTDGSHLAPLNAPLKENNKPYANKSGKVVGAYYVEWGVYGRKFT
CAD58828        NADGCTLSDKKELMVADTDGSHLAPLNAPLKENNKPYANKSGKVVGAYYVEWGVYGRKFT
ZP_01842476     NSDGCTASDAKEILVADTDGSHLVPLNAPLKENNRPYVNKSGKVVGSYFVEWGVYGRKFP
ACE78180        NADGCTASDATEIVVADTDGSHLAPLKEPLLEKNKPYKQNSGKVVGSYFVEWGVYGRNFT
BAE87051        NADGCTLSDKKELMVADTDGSHLAPLNAPLKENNKPYANKSGKVVGAYYVEWGVYGRKFT
                *.**** **  *..********* **  ** * *.** . ******.*.******** *

chi18C of SK15  VDKIPAQNLTHILYGFTPICGGNGINDSLKEISGSFEALQRSCAGREDFKVSIHDPWAAI
CAD58828        VDKIPAQNLTHILYGFTPICGGNGINDSLKEISGSFEALQRSCAGREDFKVSIHDPWAAI
ZP_01842476     VDKIPAQNLTHILYGFTPICGGDGINDSLKEIDGSFQALQRACSGREDFKVAIHDPWAAV
ACE78180        VDKIPAQNLTHLLYGFIPICGGNGINDSLKEIEGSFQALQRSCQGREDFKVSIHDPFAAL
BAE87051        VDKIPAQNLTHILYGFTPICGGNGINDSLKEISGSFEALQRSCAGREDFKVSIHDPWAAI
                ***********.**** ***** ********* ***.****.* *******.**** **.

chi18C of SK15  QMSQGNLSAWDEPYKGNFGNLMALKQAHPDLKILPSVGGWTLSDPFYFLGDKTKRDTFVA
CAD58828        QMSQGNLSAWDEPYKGNFGNLMALKQAHPDLKILPSVGGWTLSDPFYFLGDKTKRDTFVA
ZP_01842476     QMPQAGVSDYSDPYKGNFGQLMALKQAHPDLKILPSVGGWTLSDPYFYFGDKTKRDTFVA
ACE78180        QKAQKGVTAWDDPYKGNFGQLMALKQAHPDLKILPSIGGWTLSDPFFFMGDKVKRDRFVG
BAE87051        QMSQGNLSAWDEPYKGNFGNLMALKQAHPDLKILPSVGGWTLSDPFYFLGDKTKRDTFVA
                *  * ...   .*******.****************.********... *** *** **

chi18C of SK15  SVKEFLQTWKFFDGVDIDWEFPGGQGANPSLGGPNDGATYVVLMKELRAMLDELEAETGR
CAD58828        SVKEFLQTWKFFDGVDIDWEFPGGQGANPSLGGPNDGATYVVLMKELRAMLDELEAETGR
ZP_01842476     SVKEFLQTWKFFDGVDIDWEYPGGGGANPNLGNANDGETYVTLMRELRAMLDELSAETGR
ACE78180        SVKEFLQTWKFFDGVDIDWEFPGGKGANPNLGSPQDGETYVLLMKELRAMLDQLSAETGR
BAE87051        SVKEFLQTWKFFDGVDIDWEFPGGQGANPSLGGPNDGATYVVLMKELRAMLDELEAETGR
                ********************.*** **** **  .** *** **.*******.* *****

chi18C of SK15  QYELTSAISAGGDKIAKVDYQAAQQYMNHIFLMSYDFSGAFDLTNLAHQTNLFASSWDPA
CAD58828        QYELTSAISAGGDKIAKVDYQAAQQYMDHIFLMSYDFSGAFDLTNLAHQTNLFASSWDPA
ZP_01842476     TYELTSAIGIDAQKMAKVDYQAAQQYMDYIFLMSYDFNGGWTNTELGHQTNLYEASWDPD
ACE78180        KYELTSAISAGKDKIDKVAYNVAQNSMDHIFLMSYDFYGAFDLKNLGHQTALNAPAWKPD
BAE87051        QYELTSAISAGGDKIAKVDYQAAQQYMDHIFLMSYDFSGAFDLTNLAHQTNLFASSWDPA
                 *******     *. ** *. **. *  ******** *      * *** *   .* *
```

Figure S4. Nucleotide sequence of *chi*18C gene and amino acid sequence of Chi18C deduced from the gene (A) and amino acid sequence alignments of Chi18C with other chitinases (B).

(A)

```
1       GTGAATTCGAGCTCGGTACCCGGGGATCTAGGTAGTACCAGTTGCATTGCGCGAGTGATG
61      CCAAAAATAAATGAGGATGGAGACCCTTATGTCACACTTTAATCGTTTTGCTGTAGCTGC
1                   M  R  M  E  T  L  M  S  H  F  N  R  F  A  V  A  A
                                                      Signal peptide
121     AATTCCTGCTGCGCTGATGGCAGTTGCAAGCTTCTCACACGCTGCTGATGCGTGGAAAGA
18        I  P  A  A  L  M  A  V  A  S  F  S  H  A  A  D  A  W  K  E
181     AGGTAATACATATAGCGCAGGTACTGTTGTTTCATACGCTGGCCAAGACTACAAAGCTTT
38        G  N  T  Y  S  A  G  T  V  V  S  Y  A  G  Q  D  Y  K  A  L
241     GGTAACTCACACTGCCTACGTGGGTGCTAACTGGACGCCTTCTACAACTCCAACATTGTG
58        V  T  H  T  A  Y  V  G  A  N  W  T  P  S  T  T  P  T  L  W
301     GACTGCAGTTGGTGCCGCAACACCAACTACTGCACCAGTGGTAACTGCAGCGCCAACAAC
78        T  A  V  G  A  A  T  P  T  T  A  P  V  V  T  A  A  P  T  T
361     AGCGCCAGTGGTAACCGTGGCTCCAACAGCTGCGCCAGTAGTAACTAATGCGCCAACAAC
98        A  P  V  V  T  V  A  P  T  A  A  P  V  V  T  N  A  P  T  T
421     TGCACCAGTAGTGACTGCTGCACCTGGCCAAAGCTGCGGTAGTGTTCAAGCTTGGAACTC
118       A  P  V  V  T  A  A  P  G  Q  S  C  G  S  V  Q  A  W  N  S
481     AACAACTGCCTACAATGGTGGCGCGACTGTTGCGTACAACGGCAAGAAATTTACTGCTAA
138       T  T  A  Y  N  G  G  A  T  V  A  Y  N  G  K  K  F  T  A  N
541     CTGGTGGACGCAAGGCAATACGCCTTCTGCAACTGATCAGTGGGGCCCATGGAAATATGT
158       W  W  T  Q  G  N  T  P  S  A  T  D  Q  W  G  P  W  K  Y  V
601     TGGCGATTGCGCACCTGAAGTAACAGCTGCGCCAACTGCTGAACCAACTGCAACACCAGT
178       G  D  C  A  P  E  V  T  A  A  P  T  A  E  P  T  A  T  P  V
661     TGGCATGACGCCAGCGCCAACAGTTGCTCCAACTGCTGCTCCAACGCCAGTTGCTACTTT
198       G  M  T  P  A  P  T  V  A  P  T  A  A  P  T  P  V  A  T  L
721     GGCTCCAGGTCAAGAAGTTCCAGCTCCAGCAACTGCACAAGTTGGCTCTTACTTCACTCA
218       A  P  G  Q  E  V  P  A  P  A  T  A  Q  V  G  S  Y  F  T  Q
781     GTGGGGCGTTTACGGCCGTGACTACCAAGTTGCTGACATTATCAGCTCTGGTGCTGCAGC
238       W  G  V  Y  G  R  D  Y  Q  V  A  D  I  I  S  S  G  A  A  A
841     TAACTTGACGTTCATTAACTATGCATTCGGTAACATCTATCAGAAAAATGGTGGTTACGA
258       N  L  T  F  I  N  Y  A  F  G  N  I  Y  Q  K  N  G  G  Y  E
901     GTGCGGTATCGTTAACAAACTAGAACCAGGTGCAACTGATGCGAACGCTCCAGGCGCAGG
278       C  G  I  V  N  K  L  E  P  G  A  T  D  A  N  A  P  G  A  G
961     TACTGGTGGCGATGCTTGGGCTGACTTTGGTTTAACAGCTAAACGCCGTGTTGACCCTGC
298       T  G  G  D  A  W  A  D  F  G  L  T  A  K  R  R  V  D  P  A
1021    TGATCAAATCAAGTGGGACGACAAACTCGCAGGTAACTTCCGCGAATTCCAAGCGTACAA
318       D  Q  I  K  W  D  D  K  L  A  G  N  F  R  E  F  Q  A  Y  K
1081    GAAAAAATTCCCAGACACTAAACTGTTCATCTCATTGGGCGGTTGGACTTGGTCTAAGTG
338       K  K  F  P  D  T  K  L  F  I  S  L  G  G  W  T  W  S  K  W
1141    GTTCTCTGCAGCTTCGAAAACAGATGCATTGCGTAAACAGTTGGTTAGCTCTTGTATCGA
358       F  S  A  A  S  K  T  D  A  L  R  K  Q  L  V  S  S  C  I  D
1201    CATCTACATCAAAGGTAACTTGCCAGTTGTTGATGGTCGTGGTGGCCCAGGTTCAGCGCC
378       I  Y  I  K  G  N  L  P  V  V  D  G  R  G  G  P  G  S  A  P
1261    TAACATCTTCGACGGTATCGATATCGATTGGGAATTCCCAGGTGTTCAAGGCGTTGGCTA
398       N  I  F  D  G  I  D  I  D  W  E  F  P  G  V  Q  G  V  G  Y
                  Conserved region   Active site
1321    CAACACTGTAGCGCCAGAAGACAAACAAAACTTCACACTGTTGTTGGCTGAATTCCGCAA
418       N  T  V  A  P  E  D  K  Q  N  F  T  L  L  L  A  E  F  R  K
1381    ACAATTGGATGAGTTGGGTGCCGCAAACAACAAACGCTACTTGCTGACTGTGGCAATTGS
438       Q  L  D  E  L  G  A  A  N  N  K  R  Y  L  L  T  V  A  I  X
1441    TGTTGGTCGCGATAAGATCGAAATGACTGAGCCACGCGAGTACGCACGTTACCTCGACTG
458       V  G  R  D  K  I  E  M  T  E  P  R  E  Y  A  R  Y  L  D  W
1501    GATCAACATGATGACTTACGACTACAACGGTGGTTGGAATGCTCAAGGTCCTACAGACTT
478       I  N  M  M  T  Y  D  Y  N  G  G  W  N  A  Q  G  P  T  D  F
1561    CCAGTCTCACTTGTACGCTGACCCAGCTAGCCCACAATACAAGTGTTCTGGCAAAGCAGG
498       Q  S  H  L  Y  A  D  P  A  S  P  Q  Y  K  C  S  G  K  A  G
1621    CGACAAGTGCTACGGCGATCGCAGCTTGACTTCGTTCTACAACACTTCTGATGCCGTTGA
518       D  K  C  Y  G  D  R  S  L  T  S  F  Y  N  T  S  D  A  V  D
1681    CTTGCTGATCCAAGCTGGCGTGAATCCGAAGAAACTGGTTGTAGGTGTACCTAAGTACGG
538       L  L  I  Q  A  G  V  N  P  K  K  L  V  V  G  V  P  K  Y  G
1741    TCGTGGTTGGACTGGCGTAACTAACGCTAACAACGGTCTGTACCAAAAAGCGACTGATGC
558       R  G  W  T  G  V  T  N  A  N  N  G  L  Y  Q  K  A  T  D  A
1801    AGCTCGCGGCACTTACGAGAAAGGCATTGAAGACTTCAAAGTACTGAAAAACGCTGCAGG
578       A  R  G  T  Y  E  K  G  I  E  D  F  K  V  L  K  N  A  A  G
1861    TACTGTTTATGTTCACCCAGTAACTAAACAGTCTTACAAGTTTGACGGTTCTACTTTCTG
598       T  V  Y  V  H  P  V  T  K  Q  S  Y  K  F  D  G  S  T  F  W
1921    GTCTTATGACACACCAGAAGTAATCCAGACTAAGATCGACTACGCGAAAGCTAAAGGTCT
618       S  Y  D  T  P  E  V  I  Q  T  K  I  D  Y  A  K  A  K  G  L
1981    GAACGGCGGTGTATTCAGCTGGTCTCTGGACGGCGACGATAGCGCTGCAACTCTGTCTAA
638       N  G  G  V  F  S  W  S  L  D  G  D  D  S  A  A  T  L  S  K
2041    AGCAATGGGTAAAGCTCGTCAATAAGCGAGTCTAGCTCAGCAAAACCCCTCCTCGGAGGG
658       A  M  G  K  A  R  Q  *
2101    GTTTTTTTATGCCCGTCATTTATGCGAATCACTGGATGAATGGTTAAAGCACTAAGATTG
2161    GGCTGACTATACTCAAGCATTCTCCATCGCCTCGCAGGATGATGAGTCGCATGTTTCGCC
2221    GCTTTTTTGCCGACAAAGCCGCCAGCACAGAGCAAGACGCCTTTGCTCAACTTAATCAGC
2281    TTTCGCCCCTTTCCGCAGCGGAAGGGCATGAGCACGATCTGGCTCAAGCGCACGATCCGC
```

(B)

```
Chi18D of SK16  VTVAPTAAPVVTNAPTTAPVVTAAPGQSCGSVQAWNSTTAYNGGATVAYNGKKFTANWWT
AAF21468        ----PTPTPTPTPTPTPTPTPTPTPTPTPTSCF------LAWVSS-AYTGGQKVTYAGRN
ZP_02380733     ------------------PEPTPTV-----------------------------------
AAA83223        ----PTPTPTPTPTPTPEPTPTPTPTPTPSPTGGTCALAWAAGTAYSAGATVSYAGTN
AAP88583        ------------------PEPTPTV-----------------------------------
                                  *  * .

Chi18D of SK16  QGNTPSATDQWG-----------PWKYVGDCAPEVTAAPTAEPTATPVGM---TPAPTVA
AAF21468        YEAKWWTQGNVPSSN---AGEGQPWKDIGACSTTPTPTPTPTPTPTPTPTPTPTPTPTPT
ZP_02380733     ------------------------------------------------------------
AAA83223        YRANYWTQGDNPSTSSGGAGTGKPWTSQEICSTTPTPTPTPTPTPTPTPTPTPTPTPTPT
AAP88583        ------------------------------------------------------------


Chi18D of SK16  PTAAPTPVATLAPGQEVPAPATAQVGSYFTQWGVYGRDYQVADIISSGAAANLTFINYAF
AAF21468        PTPTPTPTPTPTPGAR-------QVGSYFTSGASTGATTRVKQIETSGAASKLTFLNYAF
ZP_02380733     ------------GYN---------VGSYFTQWGIYQRQYFVKNLMASGGDKKLTVLNYAF
AAA83223        PTPTPTPTPTPTGRE---------VGSYFAQWGVYGRDYEVANVHTSGVADKLTFINYAF
AAP88583        ------------GRH---------VGSYFAQWSIYGRNYKLRNLVDAGGDKKLTFLNYAF
                                        *****.          .  .  .*    ** .****

Chi18D of SK16  GNIYQKNGGYECGIVNKLEPGATDANAPGAGTGGDAWADFGLTAKRRVDPADQIKWDDKL
AAF21468        GNIYQKNGGYECDIQYRTESGNGD--------GGDPFADYSKSFSADASVDGVADKWDSP
ZP_02380733     GNVYANGQ---CGMVTAAENGNGD--------GGDAWADYQRTVSAAESVDGQADSWDTP
AAA83223        GNIYPKNGGYECGMINKAEPGATNPNSPDTGTGGDAEADYIRTPKRT--VDGQVIPWDAP
AAP88583        GNVYADGK---CGMVTRAENGNGD--------GGDAWADYQKSFGANESVDGKADSWSDP
                **.*  .    * .    * *           ***  **.  .

Chi18D of SK16  AGNFREFQAYKKKFPDTKLFISLGGWTWSKWFSAASKTDALRKQLVSSCIDIYIKGNLPV
AAF21468        LR-NFGQIKRLKARHPAISPMISLGGWSWSKWFSAASMTDPLRKQLVKSCVDLYIKQSAG
ZP_02380733     LRGNFNQLRKLKLKNPQLKVLIALGGWTWSKNFSTAAASDASRQALVSSCIDLYIKGNLP
AAA83223        LSGNFLQLKKLKAAHPNLKLFISLGGWSWSKNFSVGSATDALRKQMVRSCIDIYIKGNLP
AAP88583        LRGNFNQLRKLKLANPSLKVLISLGGWTWSKNFGKFAATDAGRKTMVASCIDLYLKGNLP
                           *            *                 .. .  .

Chi18D of SK16  VDGRGGPGSAPNI-FDGIDIDWEFPGVQGVGYNTVAPEDKQNFTLLLAEFRKQLDELGAA
AAF21468        MTAQVVRP--AAGVFDGIDIDWEFPGVQGFGYNTVSPADKQNFTLLLKEFRSQLDRCVTA
ZP_02380733     QQENAGGQGVALGVFDGFDIDWEYPVAGGLPTNHYSAADKQNFTLLLAEFRRQLDALGQQ
AAA83223        VQGGRGGVGAAANIFDGIDIDWEYPVGGGQPYNTVSPNDKRNFTLLMAEFRSQLDALGAA
AAP88583        VGENAGGAGAAKGVFDGIDIDWEYPGGGGLPSNSVDPNDKQNFTLLMAEFRSQLDALTAQ
                              *** *****.*   *   *     **.*****. *** ***

Chi18D of SK16  NN-KRYLLTVAIXVGRDKIEMTEPREYARYLDWINMMTYDYNGGWNAQ-GPTDFQSHLYA
AAF21468        TTGKRYKLTVAIGAGKDKIDMTEPAEYSKYLDWINVMSYDFHGGWDAK-GPTDFHSHLSN
ZP_02380733     NKRRYYLSAALGAGVDKIRNTEPSNYGAYLDWANVMSYDFHGGWEAQGPTNFHSNLYPDP
AAA83223        N-GKRYLLTAAVGAGKDKIDNTEPALYSQYMDWINLMTYDFHGGWETS-TNFNAQLFADP
AAP88583        NKRRYYLTAAIGSGVDKIRQTEPAKYAAYMDWINVMTYDFNGGWDAKGPTNFQSNLFRDP
                .  . *  .                  .       .      *
```

Figure S5. Nucleotide sequence of *chi*18D gene and amino acid sequence of Chi18D deduced from the gene (A) and amino acid sequence alignments of Chi18D with other chitinases (B).

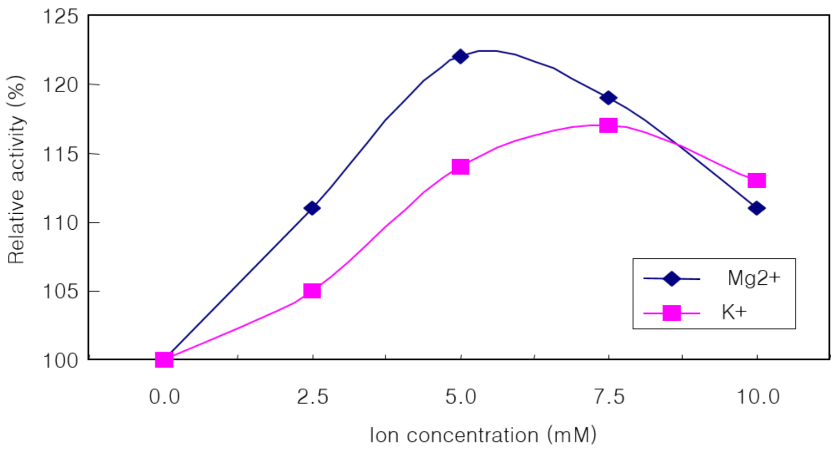

Figure S6. Effects of concentrations of Mg^{2+} and K^{+} on the enzyme activity of Chi18A.

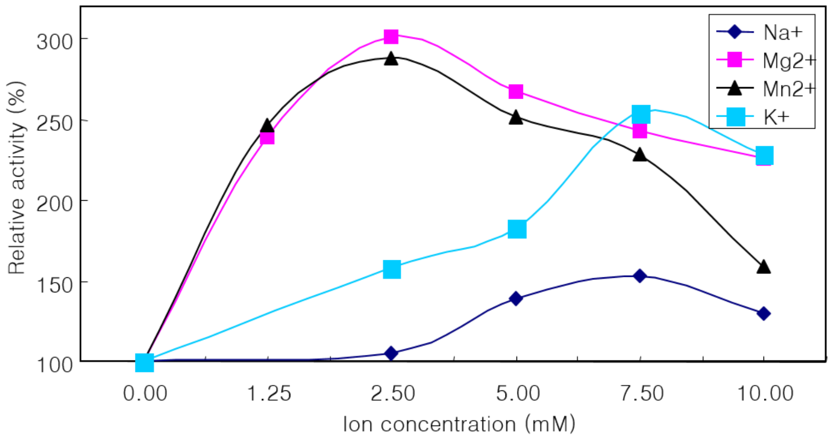

Figure S7. Effects of concentrations of Mn^{2+}, Mg^{2+}, K^{+}, and Na^{+} on the enzyme activity of Chi19B.

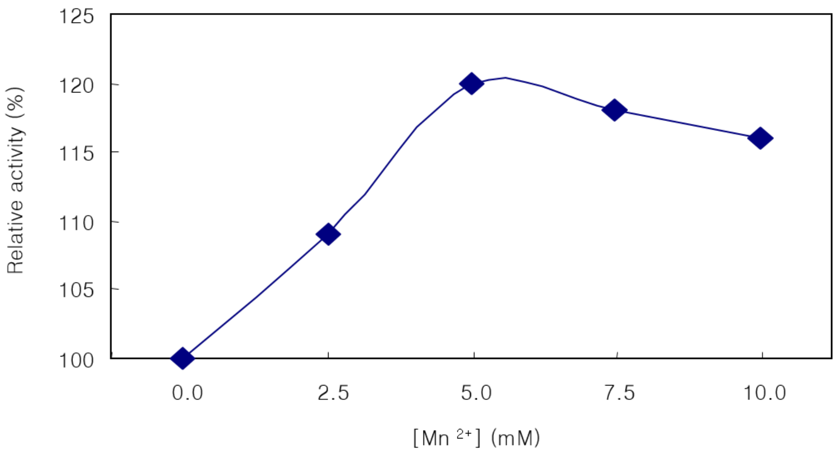

Figure S8. Effects of concentrations of Mn^{2+} (B) on the enzyme activity of Chi18D.
